# Ureido Functionalization through Amine-Urea Transamidation under Mild Reaction Conditions

**DOI:** 10.3390/polym13101583

**Published:** 2021-05-14

**Authors:** Natalia Guerrero-Alburquerque, Shanyu Zhao, Daniel Rentsch, Matthias M. Koebel, Marco Lattuada, Wim J. Malfait

**Affiliations:** 1 Laboratory for Building Energy Materials and Components, Swiss Federal Laboratories for Materials Science and Technology, Empa, Überlandstrasse 129, 8600 Dübendorf, Switzerland; natalia.guerrero@empa.ch (N.G.-A.); shanyu.zhao@empa.ch (S.Z.); matthias.koebel@empa.ch (M.M.K.); 2 Department of Chemistry, University of Fribourg, Chemin du Musée 9, 1700 Fribourg, Switzerland; marco.lattuada@unifr.ch; 3 Laboratory for Functional Polymers, Swiss Federal Laboratories for Materials Science and Technology, Empa, Überlandstrasse 129, 8600 Dübendorf, Switzerland; daniel.rentsch@empa.ch

**Keywords:** urea, amine, ureido, urease, catalyst, non-isocyanate

## Abstract

Ureido-functionalized compounds play an indispensable role in important biochemical processes, as well as chemical synthesis and production. Isocyanates, and KOCN in particular, are the preferred reagents for the ureido functionalization of amine-bearing compounds. In this study, we evaluate the potential of urea as a reagent to graft ureido groups onto amines at relatively low temperatures (<100 °C) in aqueous media. Urea is an inexpensive, non-toxic and biocompatible potential alternative to KOCN for ureido functionalization. From as early as 1864, urea was the go-to reagent for polyurea polycondensation, before falling into disuse after the advent of isocyanate chemistry. We systematically re-investigate the advantages and disadvantages of urea for amine transamidation. High ureido-functionalization conversion was obtained for a wide range of substrates, including primary and secondary amines and amino acids. Reaction times are nearly independent of substrate and pH, but excess urea is required for practically feasible reaction rates. Near full conversion of amines into ureido can be achieved within 10 h at 90 °C and within 24 h at 80 °C, and much slower reaction rates were determined at lower temperatures. The importance of the urea/amine ratio and the temperature dependence of the reaction rates indicate that urea decomposition into an isocyanic acid or a carbamate intermediate is the rate-limiting step. The presence of water leads to a modest increase in reaction rates, but the full conversion of amino groups into ureido groups is also possible in the absence of water in neat alcohol, consistent with a reaction mechanism mediated by an isocyanic acid intermediate (where the water assists in the proton transfer). Hence, the reaction with urea avoids the use of toxic isocyanate reagents by in situ generation of the reactive isocyanate intermediate, but the requirement to separate the excess urea from the reaction product remains a major disadvantage.

## 1. Introduction

Mono- and di-substituted ureas are abundant in nature and in polymer production. The ureylene functionality (R-HN-CO-NH-R) is common in natural compounds ranging from hydantoins [1,2] and diphenylureas [3] to di-alkyl ureas [4,5] and the basis of industrial polyurea production. Ureido-functionalized compounds (R-NH-CO-NH_2_) are well known for their properties and applications in medicinal and biochemistry. Ureido acids such as N-carbamoyl-β-alanine (3-ureidopropionic acid) exhibit antidiabetic properties [6]. N-Carbamoyl-glycine (hydantoic acid) is an intermediate in the formation of hydantoins [1,7,8,9], for which the derivatives fosphenytoin and phenytoin are anticonvulsants [10], whereas the derivative allantoin is well known as cancer prophylaxis or skin pro-collagen [11,12]. GABA (4-ureidobutanoic acid (N-carbamoyl-γ-aminobutyric acid) is the main inhibitory neurotransmitter in the human central nervous system [13]. Carbonylbis(L-methionine) is used in the synthesis of proinsulin analogs [14]. The derivatives of N^ε^-[N′-(2-chloroethyl)carbamoyl]-L-lysine display anti-tumoral activity [15]. Additionally, antibiotic agents such as oxazolidinone can be prepared from N-carbamoylamino alcohols [16], and N-carbamoylaminoacids are useful intermediates in the peptide synthesis [17].

Traditionally, the synthesis of ureido and ureylene materials is carried out by the reaction of amino groups with isocyanates. The most popular reagent for ureido formation is KOCN [3,7,8,9,17,18,19,20,21,22], which displays high reactivity and is used at temperatures from 20 to 100 °C but is a toxic reagent. There has been a growing interest to find non-isocyanate routes for the synthesis of polyurethane, polyurea and ureido-functionalized materials [23,24,25,26,27,28,29,30,31,32]. For non-isocyanate polyurethane synthesis, amine compounds/cyclic carbonates [33,34,35,36] and bio-based molecules [37,38,39,40] such as fatty acids [41] and vegetable oils [42,43,44,45,46] are of particular interest. For ureido and ureylene formation, urea is a potential green alternative to isocyanates [21,47]. Urea is attractive because it is a non-toxic, abundant reagent, and the synthesis can be carried out in aqueous solutions. However, the reaction of urea with amines is not widely used anymore because of the lower reactivity of urea compared to isocyanates and has not been characterized in depth.

In the past (1800s to 1940s), the transamidation of urea with amines was the default process for substituted ureas and polyurea industrial production [48,49]. The reaction was typically carried out at high processing temperatures (>120 °C) by reacting high-molecular-weight diamines or alkanolamines with urea in bulk or in solution [50,51,52,53,54,55]. These high-temperature processes are still used in industry for making products such as phenylureas [3,4,56,57,58,59,60,61] used as herbicides and coatings [61,62,63], polyalkylenureas used for melamine-formaldehyde modification [64] and cycloalkyleneureas produced from 1,2-ethylenediamine or 1,3-propylenediamine and used for easy-care cellulose-containing textiles [64,65]. Recently, ureido functionalization of chitosan, in a low-temperature aqueous process, has enabled the synthesis of high-quality chitosan aerogels [47,66]. The ureido-functionalized chitosan aerogels could even be produced by ambient pressure drying, with outstanding mechanical properties, an exceptional feat in the field of biopolymer aerogels [47].

**Scheme 1:**
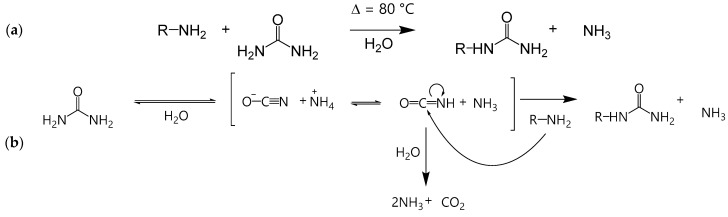

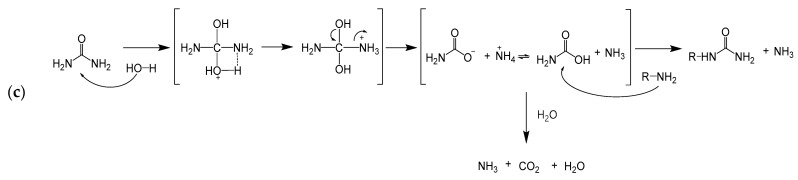
(**a**) General reaction of a primary amine with urea at 80 °C in aqueous solution to form a ureido product, (**b**) mechanism of amine with urea reaction via in situ formation of isocyanic acid in aqueous solution, and (**c**) mechanism of amine with urea reaction via urea hydrolysis.

In this study, we evaluate the potential of urea as a reagent to graft ureido groups onto amines (Scheme 1) at relatively low temperatures (<100 °C) in aqueous media.

The reaction can follow two mechanisms: in situ formation of isocyanic acid or urea hydrolysis. The isocyanate route (Scheme 1b) is considered to be the dominant mechanism when the reaction is carried out without catalysts [67,68]. The presence of water promotes Reaction B (Scheme 1b), via in situ formation of isocyanic acid (HNCO), through a hydrogen-shuttling mechanism, where the water assists the urea decomposition and favors the proton transfer between its two amino groups, promoting the formation of HNCO and NH_3_ [20,67,68]. Urea forms HNCO through the decomposition of ammonium cyanate. HNCO then undergoes carbamoylation [20], resulting in a new ureido product [21]. Alternatively, the hydrolysis of isocyanic acid into NH_3_ and CO_2_ can occur, but this is not likely because of the high reactivity of isocyanic acid with amines. In aqueous solution, urea hydrolysis (Scheme 1c) may also occur, even in the absence of a catalyst, in which case the reaction is expected to proceed through an addition–elimination mechanism [68]. A nucleophilic water attack occurs on the electrophile carbonyl carbon, followed by a tetrahedral intermediate, then the water transfers hydrogen to an amino group, ending with the release of ammonia and the formation of carbamic acid. This carbamic acid can either react with an amino group to form ureido or decompose to NH_3_ and CO_2_. Under our reaction conditions, ureido formation is expected to proceed predominantly through an isocyanic acid intermediate formed in situ from the decomposition of urea (Scheme 1b) [21,67,68]. 

In this study, we investigate the advantages and disadvantages of urea as a reagent for ureido formation for a range of substrates and synthesis parameters, including pH, solvent composition (aqueous and non-aqueous media), temperature, time, reagent concentration and molecular ratios. The reaction progress is monitored by NMR spectroscopy. We found that amino to ureido conversion close to 100% is possible in a reasonable time frame for a wide variety of amino-functionalized molecules, provided that the reaction is carried out with a large excess of urea.

## 2. Experimental Section

### 2.1. Materials

Ethanolamine ≥ 98%, hexamethylenediamine 98%, bis-(hexamethylene)triamine high purity ≥ 94%, 5-amino-1-pentanol 95%, N,N′-dimethyl-1,3-propanediamine 97%, glycine ≥ 99%, pyrrole 98%, urea BioXtra, dibutyltin dilaurate (DBTL) 95%, 1,4-diazabicyclo [2.2.2]octane (DABCO) ≥ 99%, p-toluenesulfonic acid (p-TSA) ≥ 98.5%, triethylamine (TEA) ≥ 99%, aluminum acetylacetonate (Al(acac)_3_) 99%, urease from *Canavalia ensiformis* (Jack bean), Type IX, powder, 50,000–100,000 units/g solid, 4-(2-hydroxyethyl)-1-piperazineethanesulfonic acid (HEPES) ≥ 95% (titration) and 4-morpholineethanesulfonic acid (MES) were all purchased from Sigma-Aldrich (Buchs, Switzerland). Deuterium oxide (99.9%) was obtained from Cambridge Isotope Laboratories. pH buffer solutions (pH = 4, 7, 9) for pH meter calibration were purchased from Metrohm (Herisau, Switzerland). All reagents were used without prior purification. 

### 2.2. Synthesis

Systematic parameter studies were undertaken to determine the effect of reaction conditions (reagent concentrations, pH, temperature) on the amine-to-ureido conversion. The study focuses on a series where 1 parameter is varied at a time to allow for a direct interpretation of the results and simplify the kinetic modeling, rather than a design of experiments that could find the reaction optimum with fewer experiments but results in a dataset that is harder to interpret. The substrates (Table 1) were selected to include a wide range of chemical structures but with the added criterion of low toxicity and relatively low molecular weight to ease the quantitative interpretation of the NMR data. Taking ethanolamine as a model sample, we describe a typical synthesis prepared from a 3% to 3.7% *v/v* ethanolamine solution with urea/ethanolamine in a molar ratio of 6:1. Preparations from other concentrations and molecules are summarized in Section S1 (Table S1). Typically, samples were prepared in glass vials by dissolving 2.980 g (49.6 mmol) of urea in 9 mL of distilled water and 1 mL of deuterated water (10% D_2_O was added for recording NMR with a lock on the ^2^H frequency). After urea dissolution, 0.5 mL (8.3 mmol) of ethanolamine was added to 13.2 mL of urea solution, and the samples were kept at a constant temperature (typically 80 °C or a different temperature when the effect of temperature was investigated) for 48 h under constant stirring (≈250 rpm). Aliquots were taken at different times to determine sample compositions by ^1^H and ^13^C NMR spectroscopy. For the experiments varying the pH in the solution, HCl or NaOH solutions were added until the target pH value was achieved. The synthesis experiments for the catalyst screening effort are shown in the Supplementary Materials (Section S2, Table S2). Unless explicitly stated otherwise, all data presented in this manuscript are derived from uncatalyzed experiments. 

### 2.3. Characterization

The pH was monitored at room temperature (20 °C) using pH strips with a precision of ±0.5 or using a pH meter (827 pH Lab from Metrohm (Herisau, Switzerland)) reported with a precision of 0.1 equipped with a flat-membrane electrode. The pH meter was calibrated in advance for every experiment, using a multi-point calibration with buffers of pH 4, 7 and 9 at 20 °C under stirring. After the calibration, the samples were measured twice at the same temperature and stirring conditions as the calibration. ^1^H and ^13^C NMR spectra were obtained using a 5 mm CryoProbe™ Prodigy probe equipped with z-gradient on a Bruker Advance III system with a wide-bore 9.4 T magnet, corresponding to Larmor frequencies of 400.2 MHz for ^1^H and 100.6 MHz for ^13^C. NMR samples were prepared in 10% D_2_O/H_2_O *v/v* solutions. The 1D NMR experiments were performed at 298 K using the Bruker standard pulse programs and parameter sets applying 8 (30)° pulse lengths of 1.0 (3.3) µs and relaxation delays of 4 (1) s, and 16 (512) scans were accumulated for ^1^H (^13^C) NMR data. Under these conditions, ^1^H NMR intensities are quantitative, but ^13^C NMR intensities are affected by incomplete relaxation. The sample compositions of aliquots of the reaction solution were monitored by ^1^H NMR, and the conversion was quantified by comparing the ratio of the integrated ^1^H NMR signal of the methylene group(s) adjacent to the amine (I_x_) or ureido (I_y_) group, according to Equation (1):

(1) $$NMR\;conversion=\frac{I_{x}}{I_{x}+I_{y}}$$

The NMR spectra are shown in the Supplementary Materials (Sections S3, S7 and S8).

### 2.4. Kinetic Modeling 

In order to better understand the kinetics of ureido formation, we developed a kinetic model, aiming at quantitatively accounting for the consumption of urea, the formation of ureido and the change in pH. The equations used have been reported in the Supplementary Materials (Section S9). The model takes into account all reactions in the mechanism of Scheme 1: the reversible decomposition of urea into ammonia and isocyanic acid and the reaction of isocyanic acid with and amine, as well as the decomposition of isocyanic acid to carbon dioxide and ammonia. In addition, the pH of the solution is computed by accounting for the acid–base equilibria of the amine, the ammonia, the isocyanic acid and the carbonic acid generated by the decomposition of isocyanic acid, with the caveat that pH is poorly defined in ethanol. Most of the kinetic constants for these reactions were found in the literature. The one constant that has been obtained from the experimental data is the reaction rate of isocyanic acid.

## 3. Results and Discussion

### 3.1. Ureido Formation

#### 3.1.1. Effect of the Substrate 

**Figure 1:**
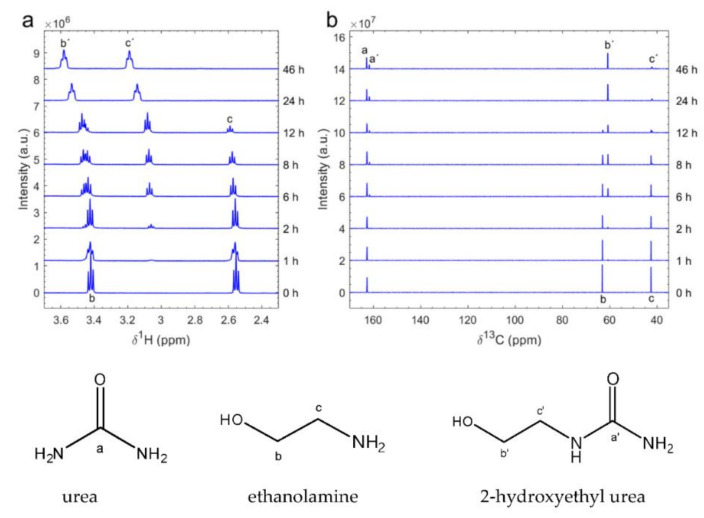
(**a**) ^1^H NMR spectra and (**b**) ^13^C NMR spectra of the formation of 2-hydroxyethyl urea from ethanolamine and urea. The reaction was conducted at 80 °C with 5% *v/v* of ethanolamine in H_2_O/D_2_O and an ethanolamine/urea ratio of 1:6. The times on the right of each spectrum indicate the reaction time. Spectra are offset vertically for clarity. Note that the ^13^C peak for c’ is broadened significantly, hence the lower peak height.

A variety of amines was studied to evaluate the feasibility of forming ureido groups by reaction with urea (Table 1). The reaction progress was followed by ^1^H and ^13^C NMR for ethanolamine (Figure 1a,b, respectively). The NMR spectra for the other substrates can be found in the Supplementary Materials (Sections S3 and S10). Both the ^1^H and ^13^C NMR indicate a progressive conversion of ethanolamine into 2-hydroxyethyl urea with increasing reaction times, with almost no detectable ethanolamine after 46 h of reaction. Because urea is used in large excess, significant quantities of unreacted urea remain in the reaction mixture. 

**Figure 2:**
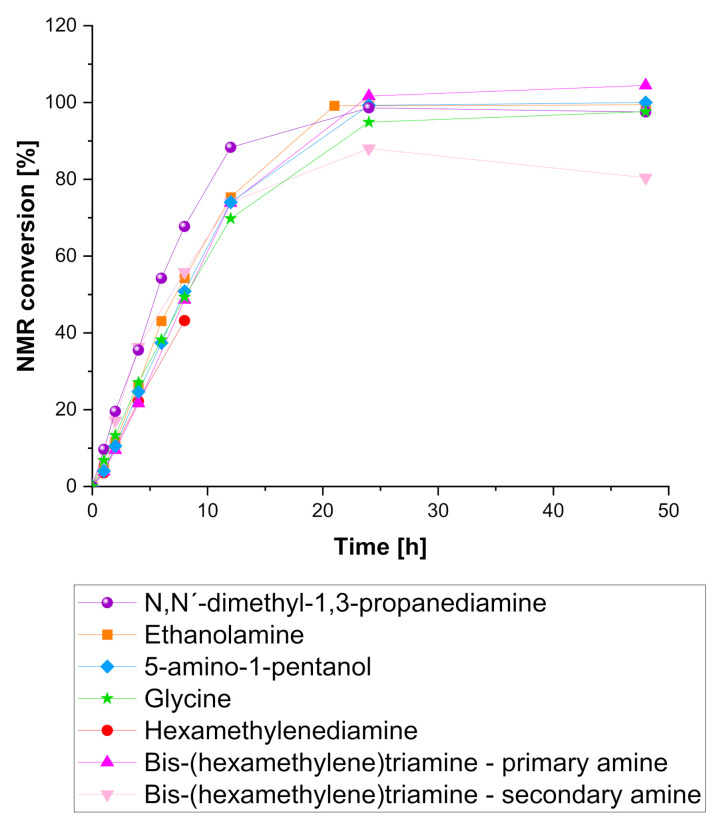
Ureido NMR conversion as a function of reaction time for different substrates. The reactions were conducted at 80 °C with 5% *v/v* of the amine molecule in H_2_O/D_2_O and with a 6-fold urea excess: ethanolamine/urea = 1:6; 5-amino-1-pentanol/urea = 1:6; hexamethylenediamine/urea = 1:6; N;N′-dimethyl-1;3-propanediamine/urea = 1:12; bis-(hexamethylene)triamine/urea= 1:18; glycine/urea = 1:6; pyrrole/urea = 1:6.

Except for pyrrole, the conversion rates (determined by ^1^H NMR) were found to be similar for all amines, independent of the substrate, with high ureido conversion (>90% after 24 h) (Figure 2, Table S1). Presumably, the lack of reactivity for pyrrole is because the nitrogen’s lone electron pair is part of the aromatic system. For hexamethylenediamine, data are only available up to 8 h because of the formation of a white precipitate for longer reaction times. Note that the reaction product of glycine and urea, hydantoic acid (n-carbamoyl-glycine), the precursor of hydantoins and derivatives [69], was also highly formed within 24 h. The effect of substrate on the reaction kinetics is modeled in detail in Section 3.5.

**Figure 3:**
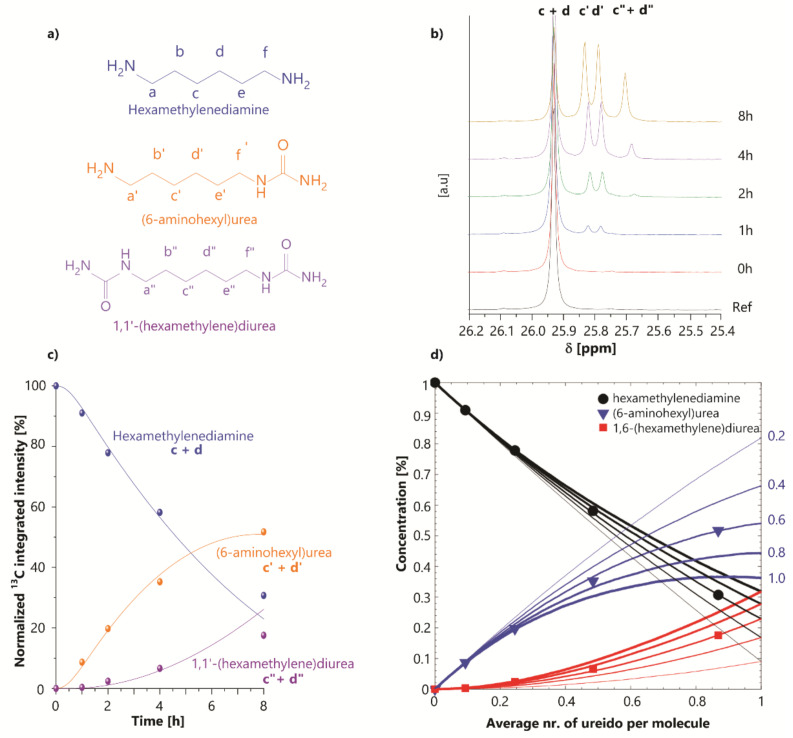
(**a**) Reactant and products of hexamethylenediamine/urea reaction. (**b**) ^13^C NMR spectra: the spectra were aligned, and the c + d resonance is located at 25.9. (**c**) NMR integrals as a function of time: the fitted lines correspond to the results of the kinetic modeling (see Section 3.5). (**d**) Experimental (markers) and modeled (lines) concentrations of hexamethylenediamine: (6-aminohexyl)urea and 1,6-(hexamethylene)diurea as a function of reaction progress. The numbers on the right denote the probability ratio for Reaction B over Reaction A (Scheme 2) (see Figure 3d) (thickest line denotes equal probabilities).

**Scheme 2:**
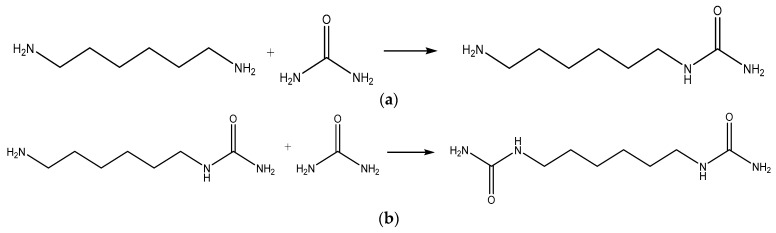
Reaction of hexamethylenediamine and urea to form: (**a**) (6-aminohexyl)urea and (**b**) 1,1-bis(6-ureidohexyl)urea.

The case of hexamethylenediamine (80 °C, 5% *v/v* hexamethylenediamine in H_2_O/D_2_O, urea/hexamethylenediamine: 12:1) is presented in more detail (Figure 3). The reaction of urea with hexamethylenediamine generates two products. First, one of the primary amines reacts with urea to yield (6-aminohexyl)urea (Scheme 2a). The remaining free amino group of this first reaction product can then react with another urea to yield 1,1′-(hexamethylene)diurea (Scheme 2b).

Thus, there are three products to take into consideration. In the ^13^C NMR spectra, the resonance at 25.92 is assigned to the midchain methylene groups at Positions C and D of the chain of the starting material The set of resonances near 25.8 ppm shifted from each other by 0.05 ppm can be attributed to the same methylenes when only one of the amines has reacted with urea to form (6-aminohexyl)urea. Here, the central methylenes (c’ and d’, Figure 3b) give rise to two peaks because the molecule is no longer symmetric. Finally, the last resonance at 25.69 ppm is assigned to the same central methylenes (c” and d”) when both amines have reacted to form 1,1′-(hexamethylene)diurea. The signal associated with the first product rapidly increases, followed by much slower increases of the signal from the second product (Figure 3c). After 8 to 12 h, the product started to precipitate, and no more aliquots were analyzed. The theoretically expected change in concentration of hexamethylenediamine, (6-aminohexyl)urea and 1,1′-(hexamethylene)diurea was predicted using Monte Carlo simulations for various probability ratios for Reaction B over Reaction A (Scheme 2) (Figure 3d, Section S4). Experimentally, more (6-aminohexyl)urea and less 1,6-(hexamethylene)diurea are formed than expected for an equal probability ratio, and the experimental results are consistent with a probability ratio of 0.6. Thus, the prior grafting of an ureido group on one end of hexamethylenediamine hinders ureido grafting on the other side of the molecule. This is also confirmed by the kinetic modeling of the NMR conversions (Section 3.5 and Section S9), from which a 50% reduction in reactivity is deduced. 

#### 3.1.2. Effect of the Temperature

**Figure 4:**
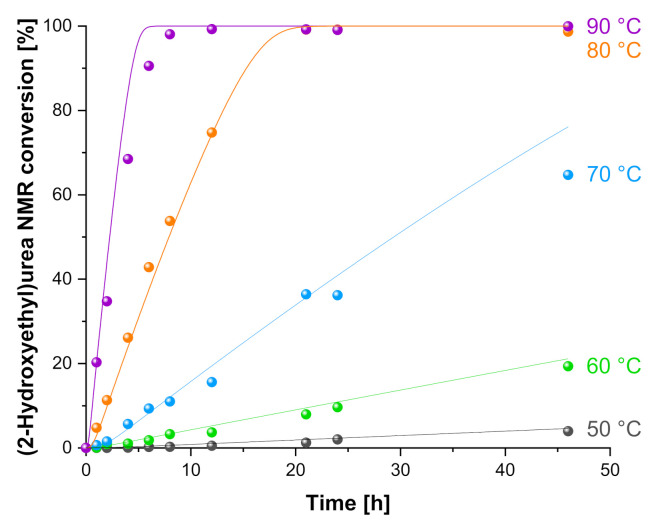
Time-dependent formation of (2-hydroxyethyl)urea as a function of reaction temperature. The reactions were conducted with 5% *v/v* of ethanolamine in H_2_O/D_2_O with a 6-fold excess of urea (ethanolamine/urea = 1:6). The fitted lines correspond to the results of the kinetic modelling (see Section 3.5).

Temperature plays an important role in the formation of the isocyanic acid and the hydrolysis of the urea. Several studies investigated amine/urea at reaction temperatures above 100 °C [21,49,55,70]. Above, we demonstrated that the reaction can be performed at lower temperatures for a wide range of substrates (Figure 2). Here, we investigate the effect of temperature more closely, using ethanolamine/urea as a model system (Figure 4, Section S7). There is a strong, non-linear effect of temperature, and a big increase in the ureido functionalization can be observed at 80 °C. At temperatures, ≥80 °C the conversion of the primary amine-to-ureido group was estimated to be >99% after 48 h. At lower temperatures, the NMR conversions are smaller and longer reaction times are required for full conversion. The effect of temperature on the reaction kinetics are modeled in detail in Section 3.5.

#### 3.1.3. Effect of Urea: Ethanolamine Ratio and pH

**Figure 5:**
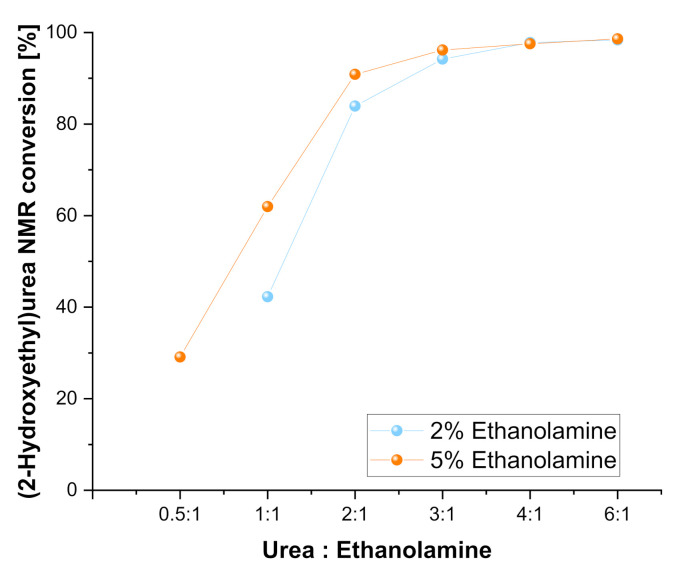
Formation of (2-hydroxyethyl)urea as a function of urea/ethanolamine ratio from 0.5:1 to 6:1. The reactions were carried out with 2% and 5% *v/v* concentrations of ethanolamine in H_2_O/D_2_O (product composition determined after 48 h at 80 °C).

Excess urea strongly increases the conversion to ureido (Figure 5). As the reaction liberates ammonia, the increased conversion for higher urea/ethanolamine ratios in theory could be an effect of pH (Table S1). However, a systematic study of the effect of pH indicates only moderate dependence of the NMR conversion on pH from 1 to 12. The NMR conversions strongly decrease for an initial pH above 12 (Section S5, Figure S7). Hence, the strong effect of the urea/ethanolamine ratio on amine-to-ureido conversion is not related to a change in pH but rather an indication that urea is the limiting reactant and, hence, urea decomposition the rate-limiting process. This is also confirmed by the kinetic modeling in Section 3.5. The need to use a large excess of urea is a major disadvantage as it complicates isolation and purification of the product after reaction.

#### 3.1.4. Effect of Ethanol as Solvent and Co-Solvent 

**Figure 6:**
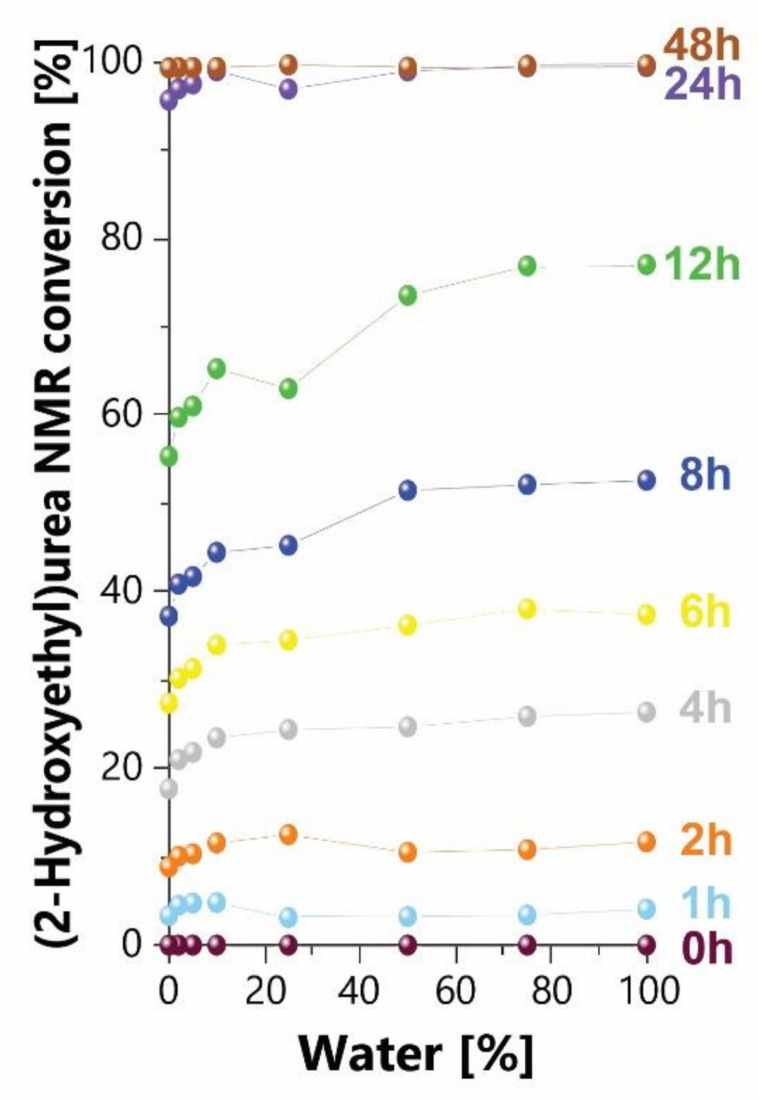
(2-hydroxyethyl)urea NMR conversion as a function of the water content of the ethanol-water mixture at different reaction times. The reactions were conducted with 5% *v/v* of ethanolamine and a 6-fold urea excess (ethanolamine/urea = 1:6) at 80 °C.

A series of experiments with different proportions of water/ethanol was conducted to evaluate the feasibility to carry out the transamidation in a non-aqueous solution rather than water (Figure 6, Section S8). The ethanolamine/urea reaction takes place even in neat ethanol, with near 100% ureido functionalization after 24 h and 80 °C, but the reaction does progress somewhat faster when the reaction is carried out in solvents with higher water content. Both the isocyanate and hydrolysis reaction mechanisms (Reactions B and C and Scheme 1) involve water. Water is obviously important for hydrolysis, and water is known to favor the formation of isocyanic acid and ammonia because of its assistance to the proton transfer between the amines of the urea [67]. The moderate dependence on water content indicates that water does promote the reaction, but it is not a requirement (as it would be for hydrolysis), consistent with an isocyanic acid intermediate as the dominant reaction mechanism under our experimental conditions.

### 3.2. Ureylene Formation

In a long-term experiment (114 days at 80 °C with an ethanolamine/urea ratio of 2:1, i.e., with a factor of two excess of ethanolamine if only ureido formation is considered), no significant amount of N,N′-bis-(hydroxyethyl)urea was observed by NMR spectroscopy: half of the ethanolamine converted into (2-hydroxyethyl)urea, but the remainder did not react, even for very long reaction times. Thus, ureylene does not form in substantial quantities under these reaction conditions, even for the longest reaction times. However, a qualitative HPLC-MS (ESI positive mode, data not shown) measurement did detect a signal at *m/z* of 149.2, consistent with protonated N,N′-bis-(hydroxyethyl)urea. Hence, the formation of traces of ureylene is likely but not in sufficient quantities to be of further interest within this study. In order to effectively form ureylene linkages, e.g., di-substituted ureas or polyurea, from (poly)amines and urea, the high-temperature processes established by the older patent studies in the literature [3,4,5,48,52,53] remain necessary.

### 3.3. Effect of Catalysts

The effect of catalysts on the reaction of ethanolamine with urea was investigated with the aim of accelerating ureido formation and, potentially catalyzing ureylene formation (Section S6). Because possible ureylene formation was a target, these catalyst experiments were carried out with an ethanolamine/urea ratio of 1:0.5, i.e., the stoichiometric ratio for ureylene formation. Note that due to this lack of excess urea, the ureido conversion is generally lower compared to the previous experiments under the same conditions but with a six-fold excess of urea (Figure 5). In these catalyst screening series, a variety of well-known catalysts from the polyurethane industry was first selected, e.g., dibutyltindilaurate (DBTL) [71], 1,4-diazabicyclo[2.2.2]octane (DABCO) [72], triethylamine (TEA) [73], aluminum acetylacetonate [74] (Al(acac)_3_) or p-toluene sulfonic acid (p-TSA) [75], where the latter is used specifically for polyurea. In the preparation of the samples, it often was difficult to find a solvent suitable for both urea and the catalyst. A mixture of 90% H_2_O and 10% D_2_O could be used for DABCO and p-TSA, but in the case of DBTL, TEA, and Al(acac)_3_, a mixture of 90% methanol (MeOH) and 10% deuterated methanol (CD_3_OD) had to be used due to the incompatibility of these catalysts in aqueous media. Because of the lower boiling point, the reaction was carried out at 60 °C, with negative effects on ureido conversion (Figure 4).

Despite the overall low NMR conversions, the direct comparison between the experiments with and without catalyst, but under otherwise identical conditions, revealed minor increases in NMR conversion with the catalyst addition to a minor extent: from 25.1% to 29.4% with DABCO, from 25.1% to 26.2% with p-TSA, from 2.2% to 13.7% with DBTL, from 2.2% to 7.65% with TEA and from 2.2% to 8.7% with Al(acac)_3_. In addition to the lackluster performance of accelerated ureido formation, no ureylene formation was observed for any of the catalysts. Thus, because of solvent compatibility issues and at the best moderate increases in ureido formation, no practical benefits could be derived from this set of catalysts.

Given the indications that urea decomposition is a rate-limiting step (Figure 5 and Section 3.5), we then turned to a catalyst that could accelerate ureido formation through the hydrolysis mechanism. Thus, urease was evaluated due to its high efficiency in promoting the hydrolysis of urea [76,77,78]. For example, urease has been applied to promote the formation of strong chitosan/urea gels [79,80]. The initial synthesis was carried out without pH adjustment, meaning high pH values around 10–11, and no catalytic activity was observed, i.e., no ureido or ureylene formation. In a second series of experiments, a buffer (HEPES) was used to adjust the pH to ranges more compatible with urease activity. This buffer was selected because it was reported to not strongly inhibit the urease activity [81]. The pH of the ethanolamine solution could be adjusted to the optimum pH of 7–8 for urease [81], but only when the buffer concentration was very high, well beyond the range where it was shown to not affect urease activity. Again, no ureido or ureylene formation was observed, most likely because of the very high buffer content. In summary, no catalysts to accelerate ureido formation or enable ureylene formation under our mild reaction conditions were identified.

### 3.4. Advantages and Disadvantages of Urea Compared to KOCN for Ureido Formation

Ureido formation from the reaction of urea with amines has distinct advantages and disadvantages. The main advantages are (i) the non-toxicity, biocompatibility, low cost and availability of urea as a feedstock; (ii) the high conversion with a wide range of amine substrates; (iii) the compatibility with water and ethanol as a solvent system; and (iv) the relatively mild reaction conditions (<100 °C, atmospheric pressure, no catalyst, wide range of pH values). These advantages are particularly relevant for chitosan aerogel production: the biopolymer aerogel field experiences a strong trend toward eliminating toxic cross-linkers, e.g., aldehydes in the case of chitosan aerogels [47,66,82]. The main downsides are (i) relatively low reaction rates (ca. 10 and 24 h) are required to reach close to 100% conversion at 80 and 90 °C, respectively, and (ii) the requirement for excess urea to accelerate the reaction, i.e., the requirement for isolation/separation after synthesis. Particularly the need for separation is a major disadvantage of the reaction. For comparison, isocyanates, with KOCN in particular, are currently the most widely used reagents to produce ureido-functionalized compounds from amines. The main advantage of KOCN over urea is the higher reactivity, hence shorter reaction times, hence the ability to carry out the reaction at temperatures between 20 and 100 °C. However, KOCN is toxic and harmful and, despite the higher reactivity, most often is also used in excess, e.g., at a KOCN/amine ratio of 2.2:1 [83], 97.1:1 [84] or 1.3:1 [85]. During the reaction with urea, toxic isocyanic acid is also formed but only in situ as a reactive intermediate. Due to the slow decomposition of urea and the high reactivity of the isocyanic acid (see below), which rapidly undergoes nucleophilic addition of amines or hydrolysis, the effective concentration of this intermediate remains low.

### 3.5. Kinetic Modeling of Uncatalyzed Reactions

The results of the kinetic modeling (Section 2.4 and Section S9) show that the reaction rate of isocyanic acid with amines is very high, several orders of magnitude higher than the rate of decomposition of urea, which is clearly the rate-determining step. The model is able to predict how the pH for most amines decreases (Figure S23), even though only slightly, during the reaction. The exception is the reaction with glycine, which, as an amino acid, starts at a low pH, with the production of ammonia, increasing it progressively. The model does a good job in quantitatively describing the conversion in ureido compounds as a function of temperature (Figure 4) and for the different substrates (Figure S24). The model also catches the major changes in pH as a function of time and temperature (Figure S25). Some deviations between model and experiment are observed for the pH, partially due to the experimental uncertainty in measured pH, and in part due to the presence of urea in solution, which, at the relatively high concentration used in the experiments, alters the ionic equilibria. This is a known effect, especially in the case of water [86], but is very difficult to be quantitatively accounted for. In addition, the glass vials were opened repeatedly to take the aliquots, enabling ammonia and CO_2_ exchange with air.

## 4. Conclusions

We demonstrate that urea is a viable alternative to isocyanates to produce ureido-functionalized compounds through a lower temperature variation of a largely forgotten high-temperature process between amines and their transamidation with urea. Urea is an inexpensive, green, non-toxic and biocompatible reagent compared to toxic isocyanates. The study revealed the possibility to synthesize ureido groups using a variety of amine substrates, including amino acids and primary and secondary amines. The reaction conditions are relatively mild: 80–90 °C, atmospheric pressure, insensitive to pH over a broad range. Excess urea is required to limit the reaction times to practically relevant durations, and the required separation is the main drawback of the reaction. The reaction rates display a strong, non-linear temperature dependence with a strong increase in reaction rates above 70 °C, consistent with urea decomposition as the rate-limiting step. The presence of water in the solvent system increases the reaction rate to a minor extent but is not essential to obtain high NMR conversions, and the reaction can also be carried with close to 100% conversion within 24 h in absolute ethanol. The modest dependence of reaction rates on water content supports the hypothesis that a mechanism with an isocyanic acid intermediate is the dominant reaction mechanism rather than a process mediated by urea hydrolysis. The transamidation reaction is similar to the standard reaction of amines with KOCN, but toxic isocyanate is generated in situ as a reactive intermediate. The use of some common catalysts moderately increased the conversion but has no practical relevance, also because of solvent/solubility compatibility issues. Even in the absence of catalysts, high NMR conversions of >95% can be achieved within 6 h at 90 °C and full conversion within 20 h at 80 °C.

## Figures and Tables

**Table 1:** Reactants, products and conversion of amine starting material after a 24 h reaction time at 80 °C.

Reactant	Product/s	Amine-to-Ureido Conversion (NMR) [%]
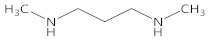 N,N′-dimethyl-1,3-propanediamine	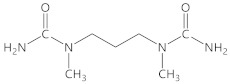 1,1′-(propane-1,3-diyl)bis(1-methylurea) 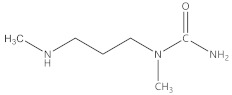 1-methyl-1-(3-(methylamino)propyl)urea	97
		3
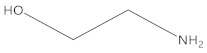 Ethanolamine	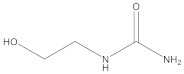 2-hydroxyethyl urea	99
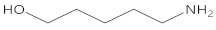 5-amino-1-pentanol	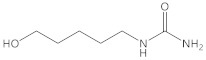 1-(5-hydroxypentyl)urea	99
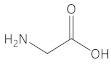 Glycine	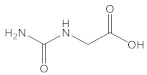 Hydantoic acid	95
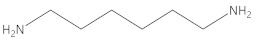 Hexamethylenediamine	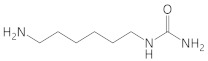 (6-aminohexyl)urea 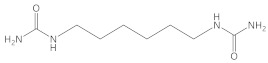 1,1′-(hexamethylene)diurea	*
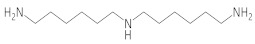 Bis-(hexamethylene)triamine	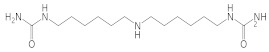 1,1′-(azanediylbis(hexane-6,1-diyl))diurea 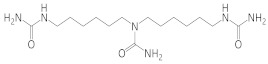 1,1-bis(6-ureidohexyl)urea	12 **
		88 **
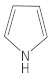 Pyrrole	-	0

* The NMR conversion at 24 h cannot be calculated from the NMR spectra because of the precipitation of the product(s). The ureido functionalization (R-NH-CO-NH_2_) was 43.2% after 8 h, in line with the other amines (Figure 2). The possibility for a continued conversion beyond 8 h of amine into ureido is corroborated by the absence of detectable unreacted hexamethylenediamine in the 24 h aliquot. ** The ureido conversion on the primary amines of bis-(hexamethylene)triamine approaches 100% at 24 h, hence possible products with one or two unreacted primary amines are not considered. The conversion of 1,1′-(azanediylbis(hexane-6,1-diyl))diurea and 1,1′-(azanediylbis(hexane-6,1-diyl))diurea can then be calculated directly from the NMR conversion of ureido modification on the secondary amine.

## Data Availability

Data is available in the Supplementary Materials and from the corresponding author on request.

## Supplementary Materials

The following are available online at https://www.mdpi.com/article/10.3390/polym13101583/s1, Figures S1–S29, Tables S1–S3.
