# Supplementary material for: Ureido Functionalization through Amine-Urea Transamidation under Mild Reaction Conditions
Source: Polymers (Basel). 2021 May 14;13(10):1583. doi: 10.3390/polym13101583 (PMC8156039; doi:10.3390/polym13101583)
Supplement: Supplementary file 1 [file polymers-13-01583-s001.zip › polymers-1184114-supplementary.pdf]

Supporting Information

Ureido-functionalization through amine-urea transamidation under mild reaction conditions

Natalia Guerrero-Alburquerque [1,2], Shanyu Zhao [1], Daniel Rentsch [3], Matthias M. Koebel [1], Marco Lattuada [2], Wim J. Malfait [1]*

[1] *Laboratory for Building Energy Materials and Components, Swiss Federal Laboratories for Materials Science and Technology, Empa, Überlandstrasse 129, 8600 Dübendorf, Switzerland*

[2] *Department of Chemistry, University of Fribourg, Chemin du Musée 9, CH-1700 Fribourg, Switzerland*

[3] *Laboratory for Functional Polymers, Swiss Federal Laboratories for Materials Science and Technology, Empa, Überlandstrasse 129, 8600 Dübendorf, Switzerland*

* corresponding author: wim.malfait@empa.ch, +41 58 765 4983

Table of content

Section S1.	Synthesis conditions of experiments without catalysts	2
Section S2.	Synthesis conditions of experiments with catalysts	8
Section S3.	NMR spectra and assignment	10
Section S4.	Information Monte Carlo simulation	17
Section S5.	Effect of pH in the reaction of ethanolamine:urea.	18
Section S6.	Catalyst screening for the ethanolamine:urea reaction	19
Section S7.	Effect of the temperature on ethanolamine:urea reaction	21
Section S8.	Effect of the solvent on ethanolamine:urea reaction	24
Section S9.	Kinetic model	29
Section S10.	^{13}C NMR spectra	36
References.		38

Section S1. Synthesis conditions of experiments without catalysts

Table S1. Experimental conditions and pH before, during and after reaction. The pH measured directly in the reactant solution, either by pH strips reported with a precision of ±0.5 or ±1; pH values measured with a pH meter reported with a precision of 0.1. For the experiments with HCl/NaOH, acid/base solution was added until the targeted pH value was reached.

Reactant [v/v]	Reactant:Urea [molar ratio]	Solvent	T [°C]	pH initial	time [h]	pH after
Ethanolamine 10%	1:0.5	D_2O	80		48	11
Ethanolamine 10%	1:1	D_2O	80		48	11
Ethanolamine 10%	1:2	D_2O	80		48	11
Ethanolamine 10%	1:3	D_2O	80		48	11
Ethanolamine 10%	1:4	D_2O	80		48	11
Ethanolamine 10%	1:6	D_2O	80		48	11
Ethanolamine 2%	1:0.5	D_2O	80		48	
Ethanolamine 2%	1:1	D_2O	80		48	
Ethanolamine 2%	1:2	D_2O	80		48	
Ethanolamine 2%	1:3	D_2O	80		48	
Ethanolamine 2%	1:4	D_2O	80		48	
Ethanolamine 2%	1:6	D_2O	80		48	
Ethanolamine 2%	1:0.5	10% D_2O/ HCl sol	80	1	48	1
Ethanolamine 2%	1:0.5	10% D_2O/ HCl sol	80	2	48	2
Ethanolamine 2%	1:0.5	10% D_2O/ HCl sol	80	3	48	3
Ethanolamine 2%	1:0.5	10% D_2O/ HCl sol	80	4	48	4
Ethanolamine 2%	1:0.5	10% D_2O/ HCl sol	80	5	48	6
Ethanolamine 2%	1:0.5	10% D_2O/ HCl sol	80	6	48	7
Ethanolamine 2%	1:0.5	10% D_2O/ NaOH sol	80	14	48	14
Ethanolamine 2%	1:0.5	10% D_2O/ NaOH sol	80	13	48	13
Ethanolamine 2%	1:0.5	10% D_2O/ NaOH sol	80	12	48	11
Ethanolamine 2%	1:0.5	10% D_2O/ NaOH sol	80	11	48	11
Ethanolamine 2%	1:0.5	10% D_2O/ NaOH sol	80	10	48	11
Ethanolamine 2%	1:0.5	10% D_2O/ NaOH sol	80	9	48	11
Ethanolamine 4.4%	1:0.5	H_2O/10% D_2O	80	10-11	1/2/3/4/5 weeks	

Ethanolamine 4.4%	1:0.5	H_2O/10% D_2O	80	11.0	0/1/2/4/8/12/24/48/72/96 h - 7/8/10/14/16/18/22/29/36/43/50/57/64/80/92/114 days	-
Ethanolamine 3.7%	1:6	H_2O/10% D_2O	90	11.5	1	10.5
					2	10.5
					4	11.5
					6	11.5
					8	11.5
					12	11.5
					24	10.0
					46	9.5
Ethanolamine 3.7%	1:6	H_2O/10% D_2O	80	11.5	1	10.5
					2	10.5
					4	11.5
					6	11.5
					8	11.5
					12	11.5
					24	10.5
					46	9.5
Ethanolamine 3.7%	1:6	H_2O/10% D_2O	70	11.5	1	10.5
					2	10.5
					4	11.5
					6	11.5
					8	11.5
					12	11.5
					24	11.5
					46	10.5
Ethanolamine 3.7%	1:6	H_2O/10% D_2O	60	11.5	1	10.5
					2	10.5
					4	11.5
					6	11.5
					8	11.5
					12	11.5
					24	11.5
					46	11.0

Ethanolamine 3.7%	1:6	H_2O/10% D_2O	50	11.5	1	10.5
					2	10.5
					4	11.5
					6	11.5
					8	11.5
					12	11.5
					24	11.5
					46	11.0
Ethanolamine 3.7%	1:6	0% EtOH pure / 100% (H_2O/10% D_2O)	80	10.5	1	10.5
					2	10.5
					4	10.5
					6	10.5
					8	10.5
					12	10.5
					24	7.5
					48	9.0
Ethanolamine 3.7%	1:6	25% EtOH pure / 75% (H_2O/10% D_2O)	80	10.5	1	10.5
					2	10.5
					4	10.5
					6	10.5
					8	10.5
					12	10.5
					24	9.0
					48	9.0
Ethanolamine 3.7%	1:6	50% EtOH pure / 50% (H_2O/10% D_2O)	80	10.5	1	10.5
					2	10.5
					4	10.5
					6	10.5
					8	10.0
					12	10.0
					24	9.5
					48	9.0

Ethanolamine 3.7%	1:6	75% EtOH pure / 25% (H_2O/10% D_2O)	80	10.5	1	9.5
					2	9.5
					4	9.5
					6	9.5
					8	9.5
					12	10.0
					24	9.0
					48	9.0
Ethanolamine 3.7%	1:6	90% EtOH pure / 10% D_2O	80	10.5	1	9.5
					2	9.5
					4	9.5
					6	9.5
					8	9.5
					12	10.0
					24	9.0
					48	9.0
Ethanolamine 3.7%	1:6	100% EtOH pure / 0% D_2O (Addition of D_2O after the reaction)	80	7	1	7.5
					2	7.5
					4	7.7
					6	7.8
					8	7.9
					12	8.1
					24	8.4
					48	8.7
Ethanolamine 3.7%	1:6	98% EtOH pure / 2% D_2O (Addition of D_2O after the reaction)	80	-	1	11.0
					2	-
					4	-
					6	-
					8	-
					12	-
					24	-
					48	-

Ethanolamine 3.7%	1:6	95% EtOH pure / 5% D_2O (Addition of D_2O after the reaction)	80	-	1	11.0
					2	11.7
					4	11.7
					6	11.7
					8	11.6
					12	11.6
					24	11.3
					48	11.2
Ethanolamine 3.7%	1:6	90% EtOH pure / 10% D_2O (Addition of D_2O after the reaction)	80	10.5	1	11.0
					2	11.8
					4	11.7
					6	11.7
					8	11.6
					12	11.6
					24	11.3
					48	11.2
Hexamethylenediamine 3.6%	1:12	H_2O/10% D_2O	80	13	1	12.5
					2	12.5
					4	12.0
					8	12.0
					12	12.0
					24	10.0
					48	10.0
Bis-(Hexamethylene)triamine 3.9%	1:18	H_2O/10% D_2O	80	13	1	12.5
					2	12.5
					4	12.0
					8	12.0
					12	11.5
					24	10.0
					48	10.0
5-amino-1-pentanol 4.1%	1:6	H_2O/10% D_2O	80	11.5	1	12.0
					2	12.0
					4	11.9
					8	11.7
					12	11.4
					24	10.6
					48	10.3

N,N'-Dimethyl-1,3-propanediamine 3.6%	1:6	H_2O/10% D_2O	80	12.5	1	12.5
					2	12.2
					4	12.0
					8	11.7
					12	11.3
					24	10.8
					48	10.5
Glycine 2.6%	1:6	H_2O/10% D_2O	80	7	1	7.5
					2	7.5
					4	7.7
					8	7.9
					12	8.0
					24	8.3
					48	8.6
Pyrrole 3.8%	1:6	H_2O/10% D_2O	80	-	-	-

Section S2. Synthesis conditions of experiments with catalysts

Table S2. Synthesis conditions for the experiments with catalysts: reactant, buffer and catalyst concentrations, solvent composition, pH, temperature and time. The reference pH corresponds to the pH value of the solutions before adding the catalyst. The pH measured directly in the reactant solution, either by pH strips reported with a precision of ±0.5 or ±1; pH values measured with a pH meter reported with a precision of 0.1.

Reactant [v/v]	Reactant:Urea [molar ratio]	Catalyst [mg]	Solvent	Solution [mL]	HEPES [M]	Ref. pH_i-pH_f	pH_i	pH_f	T [°C]	time [h]
Ethanolamine 5%	1:0.5	Urease 0.45	H_2O/10% D_2O	2	-		10	10	37	24
Ethanolamine 0.5%	1:0.5	Urease 0.19	HEPES H_2O / 10% D_2O	5	0.5	6.5-7	6.5	7	37	24
Ethanolamine 0.5%	1:0.5	Urease 0.38	HEPES H_2O / 10% D_2O	5	0.5	6.5-7	6.5	7/8*	37/50 *	24/24 *
Ethanolamine 0.5%	1:0.5	Urease 0.56	HEPES H_2O / 10% D_2O	5	0.5	6.5-7	6.5	7/8*	37/50 *	24/24 *
Ethanolamine 0.5%	1:0.5	Urease 0.75	HEPES H_2O / 10% D_2O	5	0.5	6.5-7	6.5	7/8*	37/50 *	24/24 *
Ethanolamine 1.5%	1:0.5	Urease 2.26	HEPES H_2O / 10% D_2O	5	0.5	8-8	8	8.5	37	24
Ethanolamine 1.5%	1:0.5	Urease 5.65	HEPES H_2O / 10% D_2O	5	0.5	8-8	8.5	9	37	24
Ethanolamine 1.5%	1:0.5	Urease 5.65	H_2O/ 10% D_2O	5	-	11-11	11	10	37	24
Ethanolamine 1.5%	1:0.5	Urease 5.65	HEPES H_2O / 10% D_2O	5	0.5	8-8	8	8.5	80	24
Ethanolamine 5%	1:0.5	DBTL 11.31	MeOH/10% CD_3OD	5					60	24/48
Ethanolamine 5%	1:0.5	DBTL 3.76	MeOH/10% CD_3OD	5					60	24/48
Ethanolamine 5%	1:0.5	DBTL 1.13	MeOH/10% CD_3OD	5					60	24/48
Ethanolamine 5%	1:0.5	DBTL 0.0	MeOH/10% CD_3OD	5					60	24/48
Ethanolamine 5%	1:0.5	DABCO 11.31	H_2O/10% D_2O	5					80	24/48

Ethanolamine 5%	1:0.5	DABCO 3.76	H_2O/10% D_2O	5	80	24/48
Ethanolamine 5%	1:0.5	DABCO 1.13	H_2O/10% D_2O	5	80	24/48
Ethanolamine 5%	1:0.5	DABCO 0	H_2O/10% D_2O	5	80	24/48
Ethanolamine 5%	1:0.5	p-TSA 11.31	H_2O/10% D_2O	5	80	24/48
Ethanolamine 5%	1:0.5	p-TSA 3.76	H_2O/10% D_2O	5	80	24/48
Ethanolamine 5%	1:0.5	p-TSA 1.13	H_2O/10% D_2O	5	80	24/48
Ethanolamine 5%	1:0.5	p-TSA 0	H_2O/10% D_2O	5	80	24/48
Ethanolamine 5%	1:0.5	TEA 11.31	MeOH/10% CD_3OD	5	60	24/48
Ethanolamine 5%	1:0.5	TEA 3.76	MeOH/10% CD_3OD	5	60	24/48
Ethanolamine 5%	1:0.5	TEA 1.13	MeOH/10% CD_3OD	5	60	24/48
Ethanolamine 5%	1:0.5	TEA 0	MeOH/10% CD_3OD	5	60	24/48
Ethanolamine 5%	1:0.5	$Al(acac)_3$ 11.31	MeOH/10% CD_3OD	5	60	24/48
Ethanolamine 5%	1:0.5	$Al(acac)_3$ 3.76	MeOH/10% CD_3OD	5	60	24/48
Ethanolamine 5%	1:0.5	$Al(acac)_3$ 1.13	MeOH/10% CD_3OD	5	60	24/48
Ethanolamine 5%	1:0.5	$Al(acac)_3$ 0	MeOH/10% CD_3OD	5	60	24/48

**After the first 24h, the temperature of the reaction was increased to 50 °C and it was kept additionally at this temperature another 24h.*

Section S3. NMR spectra and assignment

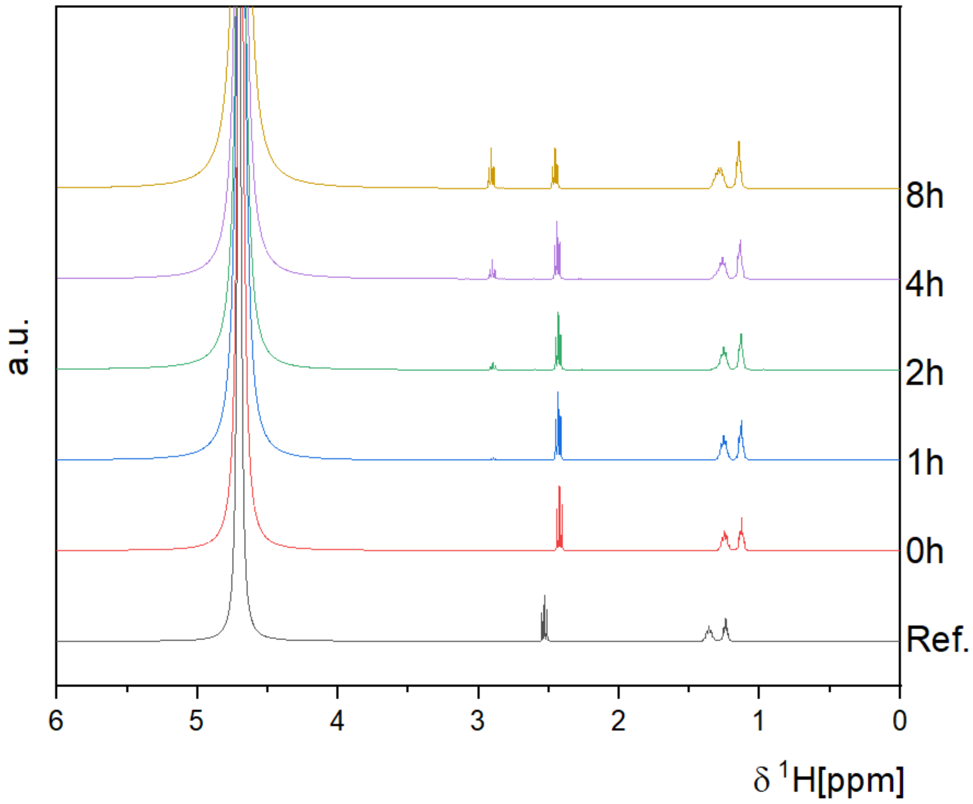

hexamethylenediamine	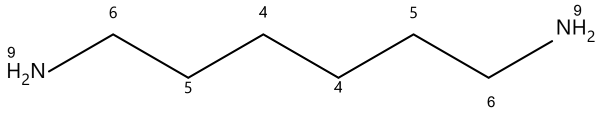
(6-aminohexyl)urea	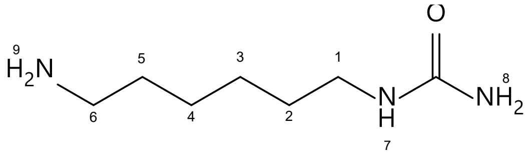
1,1'-(hexamethylene)diurea	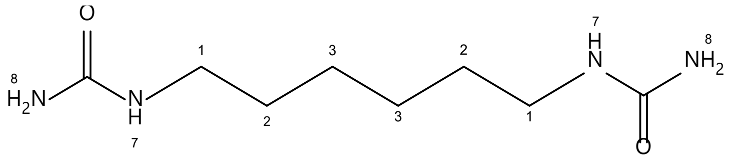

Figure S1. ¹H NMR spectra and numbering of positions of the products of hexamethylenediamine after reaction with urea. The reaction was conducted at 80 °C with 5%v/v of hexamethylenediamine in H_2O/D_2O and with a 12-fold urea excess (hexamethylenediamine:urea 1:12).

¹H NMR parameters derived from the spectrum collected after 8 h of reaction. The peak positions shift at different reaction times because of changes in pH, hence, only the peak positions relative to each other are considered relevant: ¹H-NMR (400.2 MHz, 10% D_2O/H_2O, 298K): δ= 2.91 (*t, J=7.0,* 2H, H1), 2.45 (*t, J=7.7*, 2H, H6), 1.28 (*m,* 8H, H2,H5), 1.15 (*m,* 4H, H3&H4).

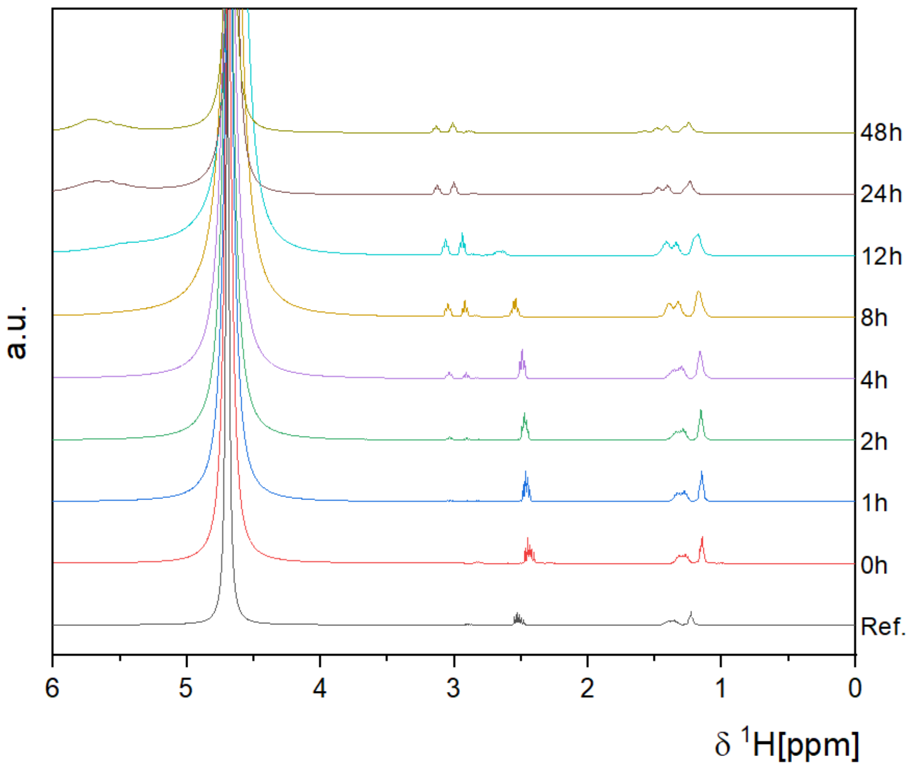

bis-(hexamethylene) triamine	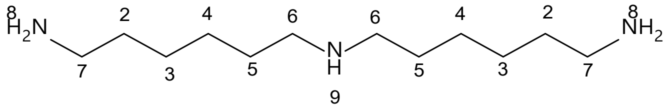
1,1-bis(6-aminohexyl)urea	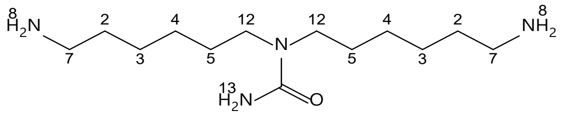
1-(6-(6-aminohexyl)aminohexyl)urea	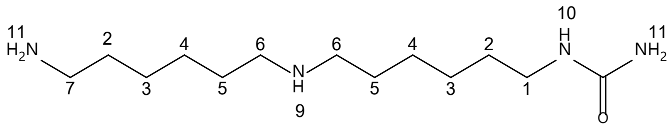
1-(6-aminohexyl)-1-(6-ureidohexyl)urea	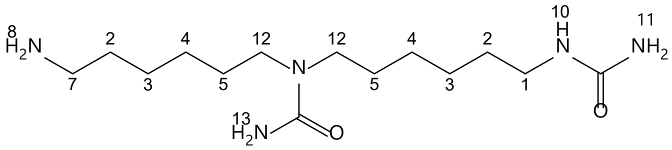
1,1'-(azanediylbis(hexane-6,1-diyl))diurea	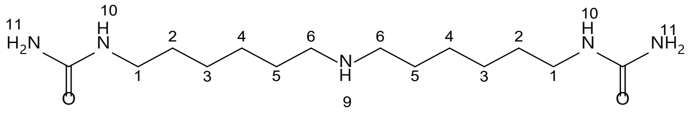
1,1-bis (6-ureidohexyl)urea	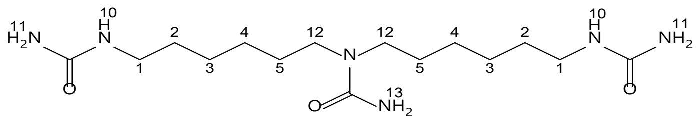

Figure S2. ^{1}H NMR spectra and numbering of positions of the possible reaction products of bis-(hexamethylene)triamine with urea. The reaction was conducted at 80 °C with 5%v/v of bis-(hexamethylene)triamine in H_2O/D_2O and with a 18-fold urea excess with respect to the urea:amine ratio, corresponding to (bis-(hexamethylene)triamine:urea 1:18). Note that the H2, H3, H4 and H5 protons of the possible reaction products all overlap into single multiplets.

^{1}H NMR parameters derived from the spectrum collected after 24 h of reaction. The peak positions shift at different reaction times because of changes in pH, hence, only the peak positions relative to each other are considered relevant: ^{1}H-NMR (400.2 MHz, 10% D_2O/H_2O, 298K): δ= 3.12 (*t, J=7.2*, 4H, H12), 3.00 (*t, J=6.4*, 4H, H1), 2.85 (*t, J= 7.7*, impurity), 1.56 (*t, J=6.9*, 4H, H5), 1.47 (*t, J=6.1*, 4H, H2), 1.40 (*t, J=6.5*, 4H, H3), 1,24 (s broad, 4H, H4).

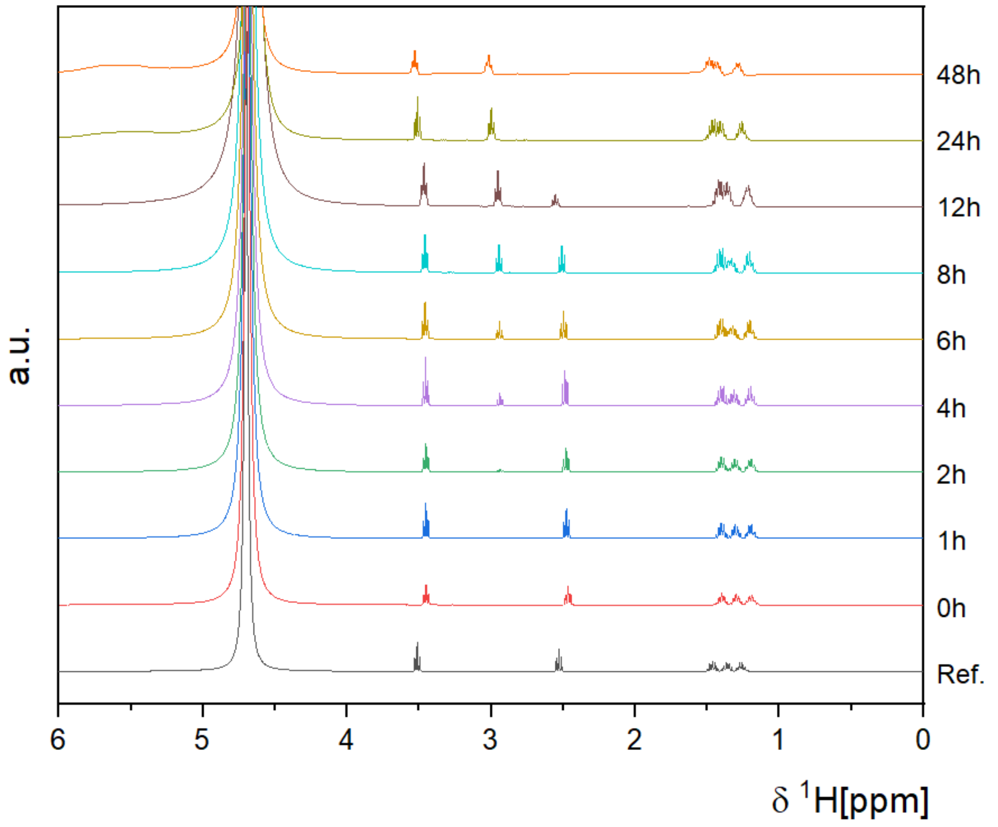

5-amino-1-pentanol	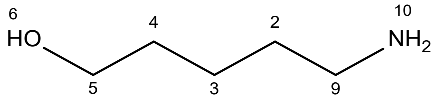
1-(5-hydroxypentyl)urea	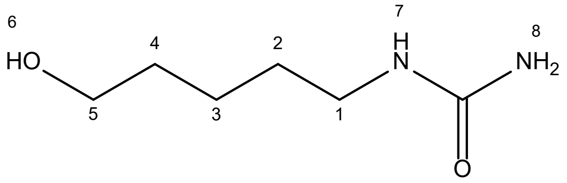

Figure S3. *^{1}H NMR spectra and numbering of positions of the possible reaction products of 5-amino-1-pentanol after reaction with urea. The reaction was conducted at 80 °C with 5%v/v of 5-amino-1-pentanol in H_2O/D_2O and with a 6-fold urea excess (5-amino-1-pentanol:urea 1:6).*

^{1}H NMR parameters derived from the spectrum collected after 24 h of reaction. The peak positions shift at different reaction times because of changes in pH, hence, only the peak positions relative to each other are considered relevant: ^{1}H-NMR (400.2 MHz, 10% D_2O/H_2O, 298K): δ= 3.51(*t, J=6.57*, CH_2, 2H, H5), 2.99(*t, J=6.89*, CH_2, 2H, H1), 2.75 (*t, J= 7.45*, CH_2, 2H, H9), 1.46 (*quint, J=7.12*, CH_2, 2H, H4), 1.41(*quint, J=7.43*, CH_2, NH_2, 2H, H2,H7), 1.25 (*quint, J= 8.02*, CH_2, 2H, H3)

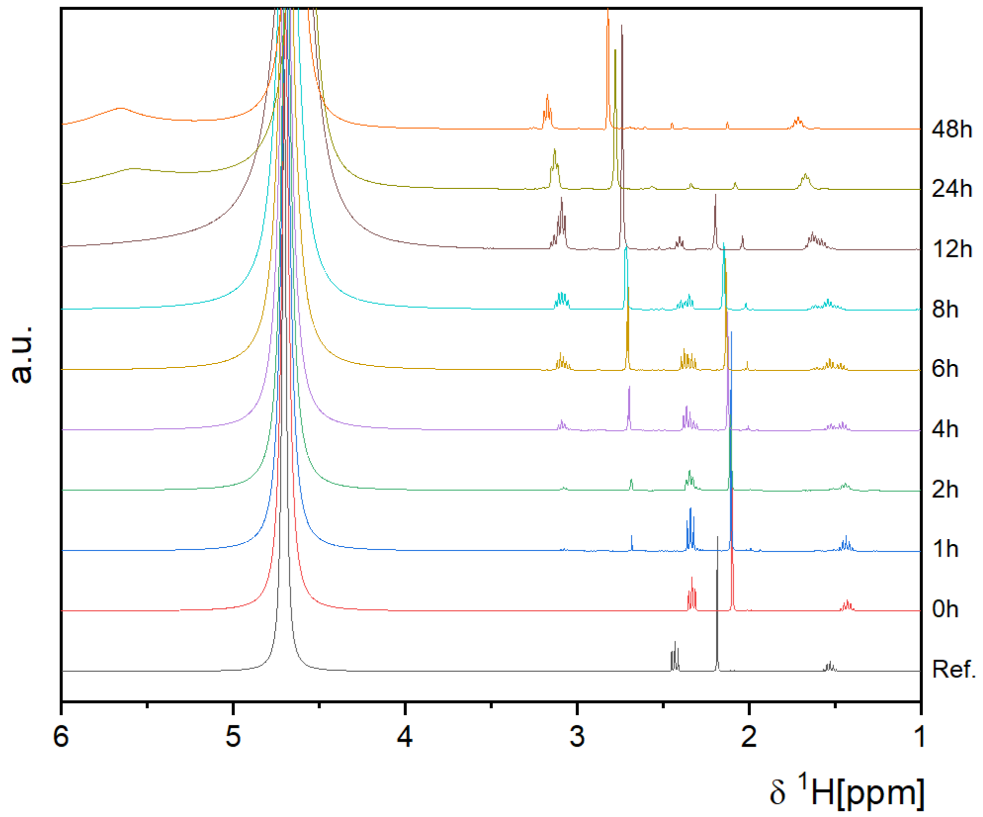

n,n'-dimethyl-1,3-propanediamine	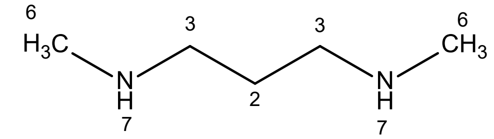
1-methyl-1-(3-(methylamino)propyl)urea	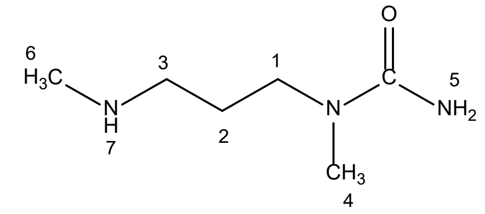
1,1'-(propane-1,3-diyl)bis(1-methylurea)	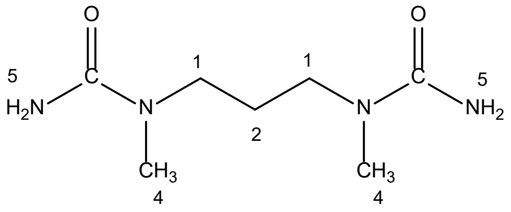

Figure S4. ^{1}H NMR spectra and numbering of positions of the possible reaction products of N,N'-dimethyl-1,3-propanediamine after reaction with urea. The reaction was conducted at 80 °C with 5%v/v of N,N'-dimethyl-1,3-propanediamine in H_2O/D_2O and with a 12-fold urea excess (N,N'-dimethyl-1,3-propanediamine:urea 1:12).

^{1}H NMR parameters derived from the spectrum collected after 24 h of reaction. The peak positions shift at different reaction times because of changes in pH, hence, only the peak positions relative to each other are considered relevant: ^{1}H-NMR (400.2 MHz, 10% D_2O/H_2O, 298K): δ= 3.16(*t, J= 7.47*, CH_2, 2H, H1), 2.80 (*s*, CH_3, 3H, H4&H6), 2.34 (*s*, CH_2, 2H, H3), 1.70 (*quint, J=7.31*, CH_2, 2H, H2)

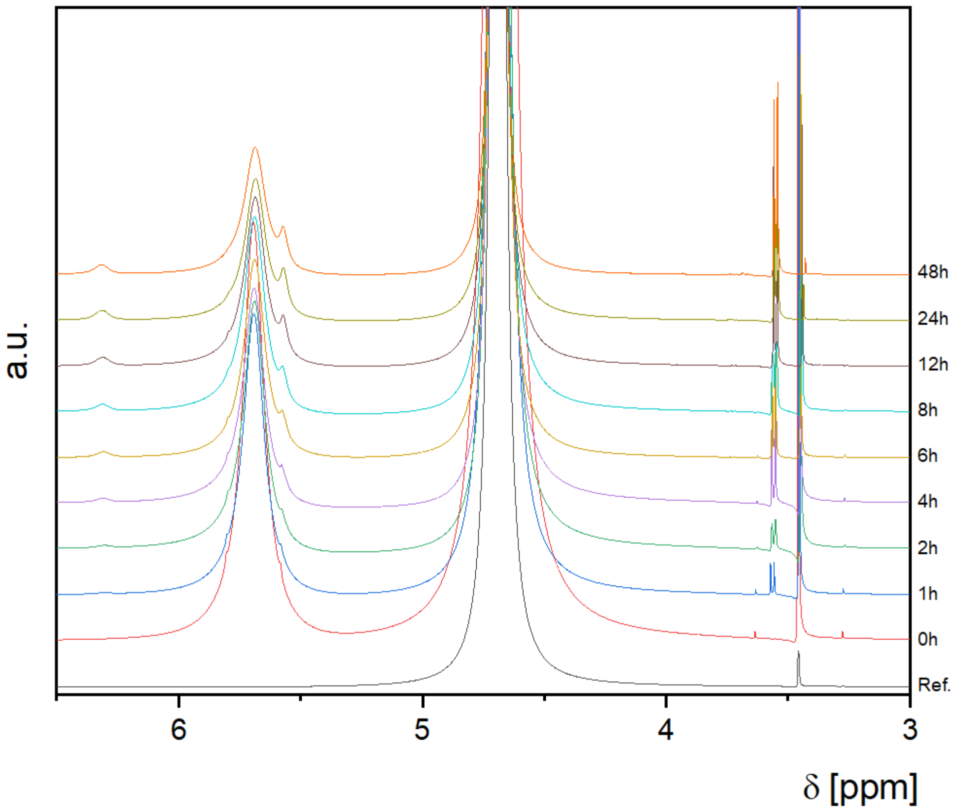

glycine	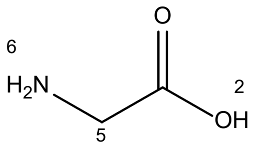
hydantoic acid	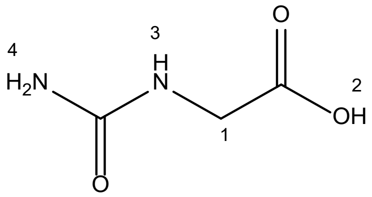

Figure S5. ^{1}H NMR spectra and numbering of positions of the possible reaction products of glycine after reaction with urea. The reaction was conducted at 80 °C with 5%v/v of glycine in H_2O/D_2O and with a 6-fold urea excess (glycine:urea 1:6).

^{1}H NMR parameters derived from the spectrum collected after 24 h of reaction. The peak positions shift at different reaction times because of changes in pH, hence, only the peak positions relative to each other are considered relevant: ^{1}H-NMR (400.2 MHz, 10% D_2O/H_2O, 298K): δ= 6.33 (*s*, NH, 1H, H3), 3.54 (*d*, *J*= *5.91*, 2H, H1), 3.43 (*s*, 2H, H5). The resonance of H-1 at δ= 3.54 is split into a doublet due to the coupling with H-3 of the ureido group, which under the measuring conditions shows slow chemical exchange with water (broadening of NH resonance at 6.33 ppm). In a ^{1}H-^{1}H DQF-COSY NMR spectrum the expected cross peak of H-1 to the NH proton was clearly observed.

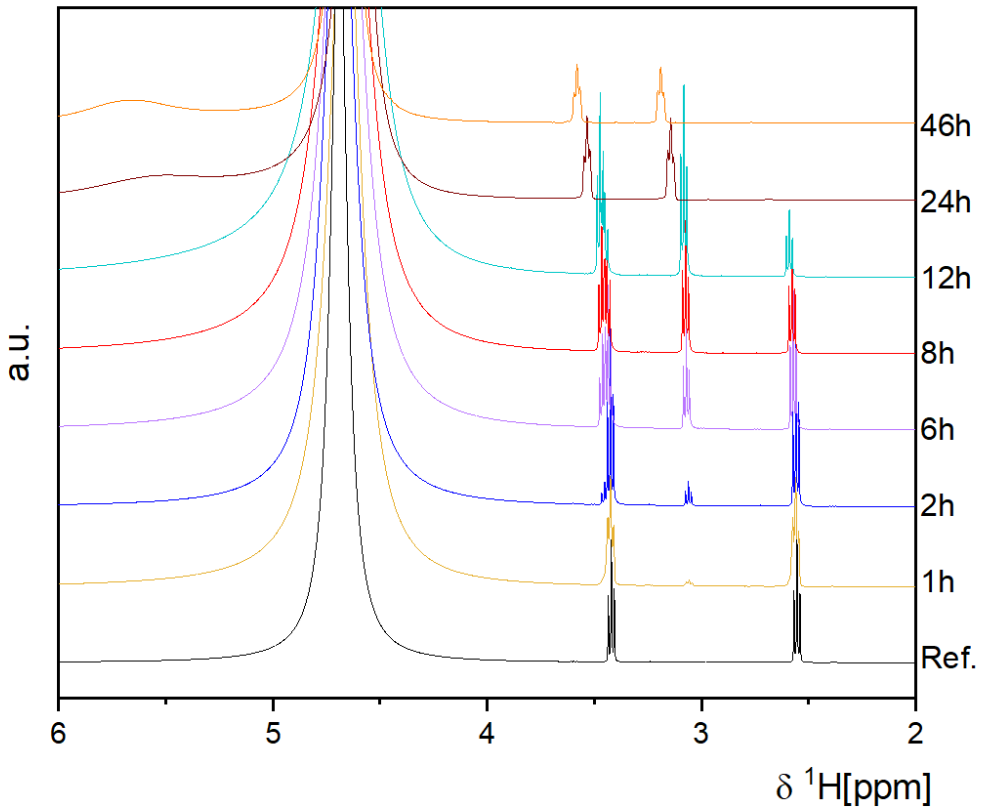

ethanolamine	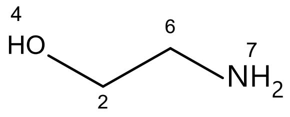
2-hydroxyethyl urea	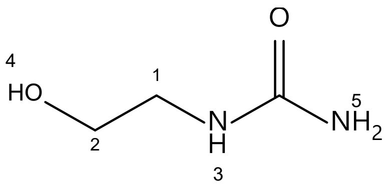

Figure S6. ^{1}H NMR spectra and numbering of positions of the possible reaction products of ethanolamine after reaction with urea. The reaction was conducted at 80 °C with 5%v/v of ethanolamine in H_2O/D_2O and with a 6-fold urea excess (ethanolamine:urea 1:6)

^{1}H NMR parameters derived from the spectrum collected after 24 h of reaction. The peak positions shift at different reaction times because of changes in pH, hence, only the peak positions relative to each other are considered relevant: ^{1}H-NMR (400.2 MHz, 10% D_2O/H_2O, 298K): δ=, 3.52 (*t, J= 5.56*, 2H, H2), 3.15 (*t, J= 5.50*, 2H, H1), 2.65(*t, J= 5.61*, 2H, H6)

Section S4. Information Monte Carlo simulation

Matlab code for Monte Carlo simulation of expected concentrations of hexamethylenediamine, (6-aminohexyl)urea and 1,6-(hexamethylene)diurea as a function of reaction progress.

```
a0start=100000;               % number of hexamethylenediamine molecules
a0=a0start; a1=0; a2=0;       % starting concentrations
for i=1:2*a0start
    if rand<1.0*a1/(a1+a0)    % set probability to calculate
        a1=a1-1; a2=a2+1;     % conversion of a1 into a2
    else
        a0=a0-1; a1=a1+1;     % conversion of a0 into a1
    end
    a0s(i)=a0; a1s(i)=a1; a2s(i)=a2;     % log concentrations
end
pma=(a1s+2*a2s)/a0start;      % calculate average reaction progressio
```

Section S5. Effect of pH in the reaction of ethanolamine:urea

The effect of pH on the ethanolamine:urea (ratio 1:0.5) reaction to form (2-hydroxyethyl)urea was studied after 48h hours at 80 °C in solution. Experiments were carried out after adjustment of the initial pH with either HCl or NaOH solutions, depending on the target pH.

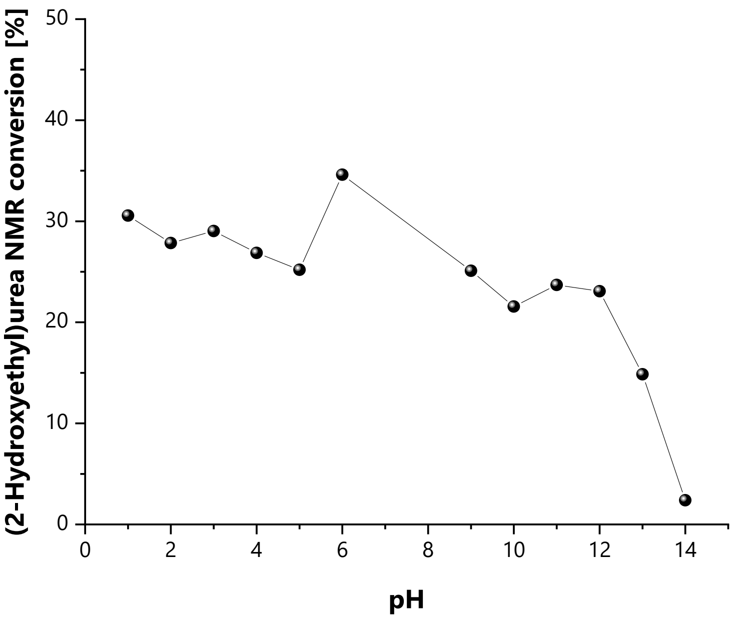

Figure S7. pH study of (2-hydroxyethyl)urea NMR conversion as a function of pH. The reaction with 2% v/v of ethanolamine in H_2O/D_2O, and ethanolamine:urea ratio of 1:0.5 were conducted at 80 °C for 48h.

Section S6. Catalyst screening for the ethanolamine:urea reaction

Group 1: Water compatible catalysts.

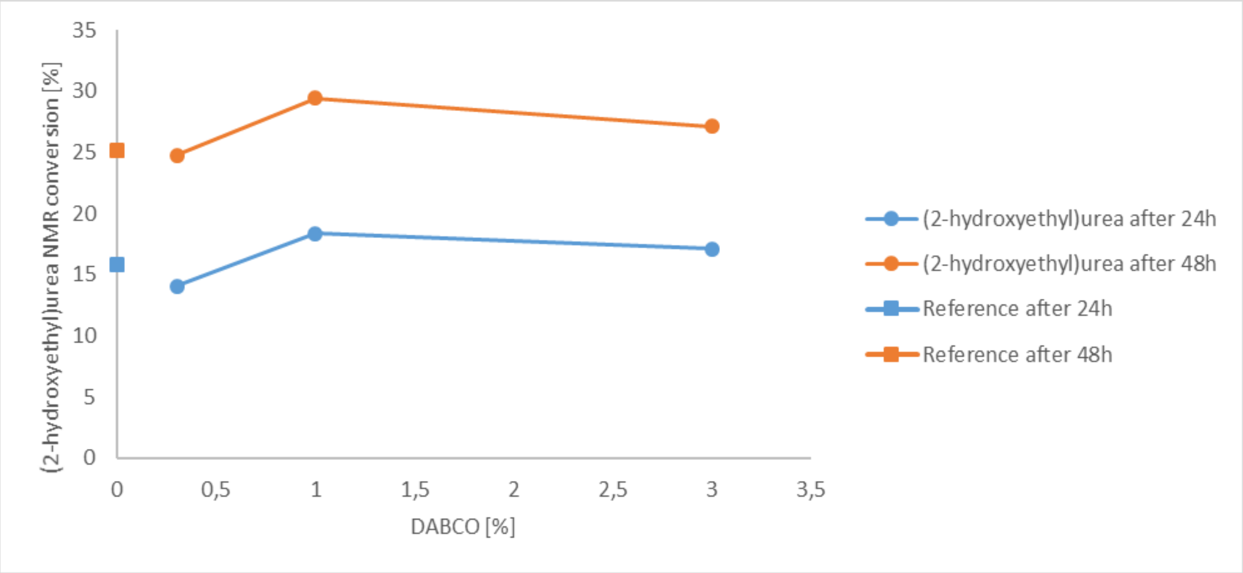

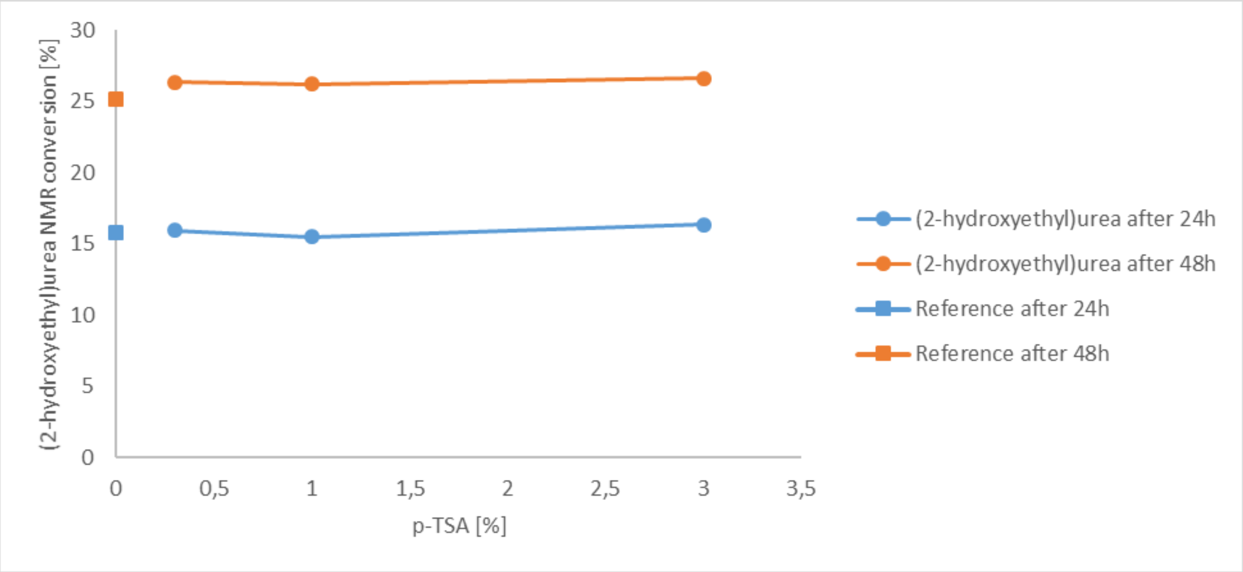

Figure S8. NMR conversion as a function of catalyst concentration. Group 1: The reactions were conducted with 5% v/v ethanolamine in H_2O/D_2O at 80 °C (ethanolamine:urea 1:0.5)

Group 2: Water incompatible catalysts: reaction in methanol.

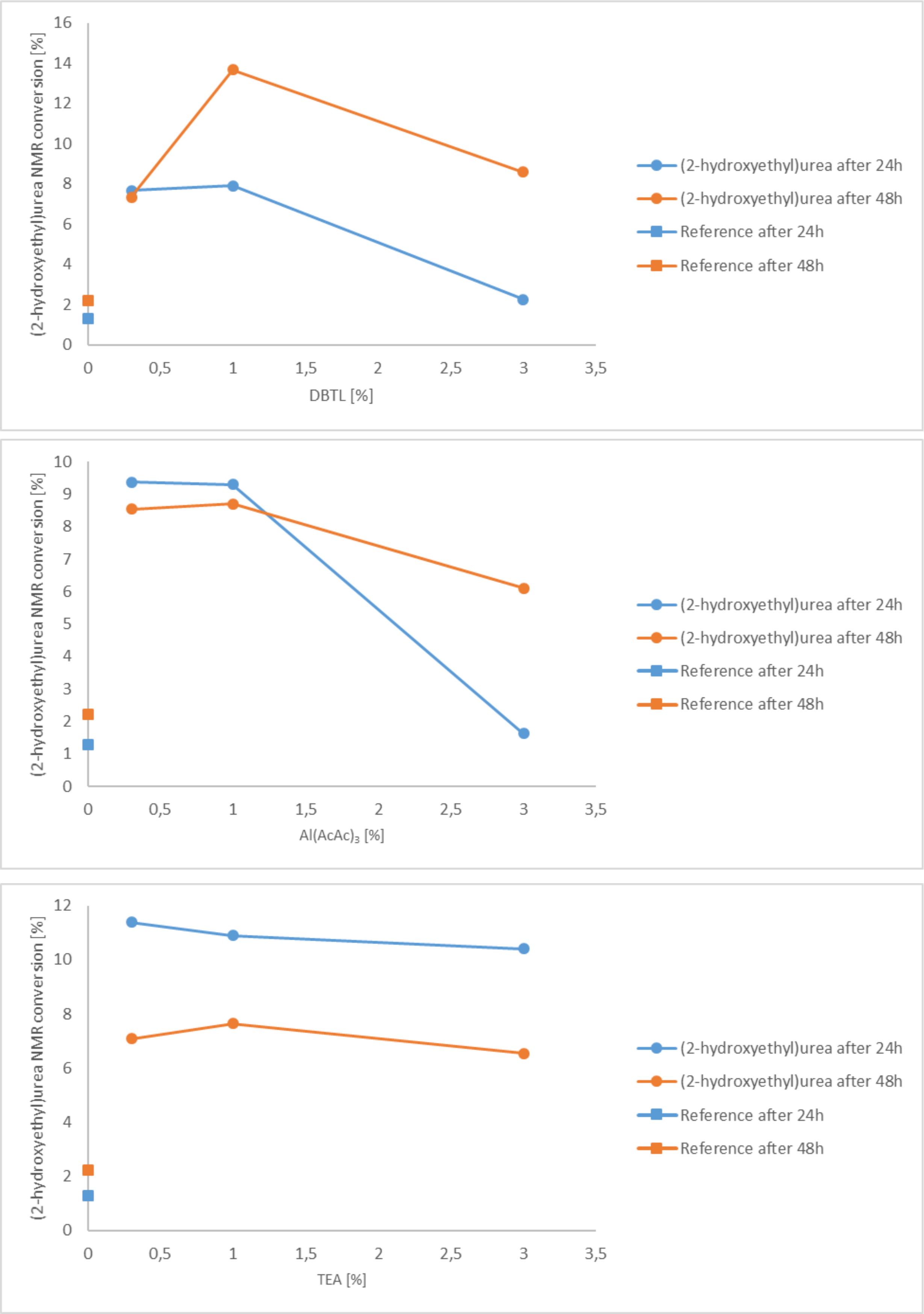

Figure S9.NMR conversion as a function of catalyst concentration. Group 2: The reactions were conducted with 5% v/v ethanolamine in methanol at 60 °C (ethanolamine:urea 1:0.5)

Section S7. Effect of the temperature on ethanolamine:urea reaction

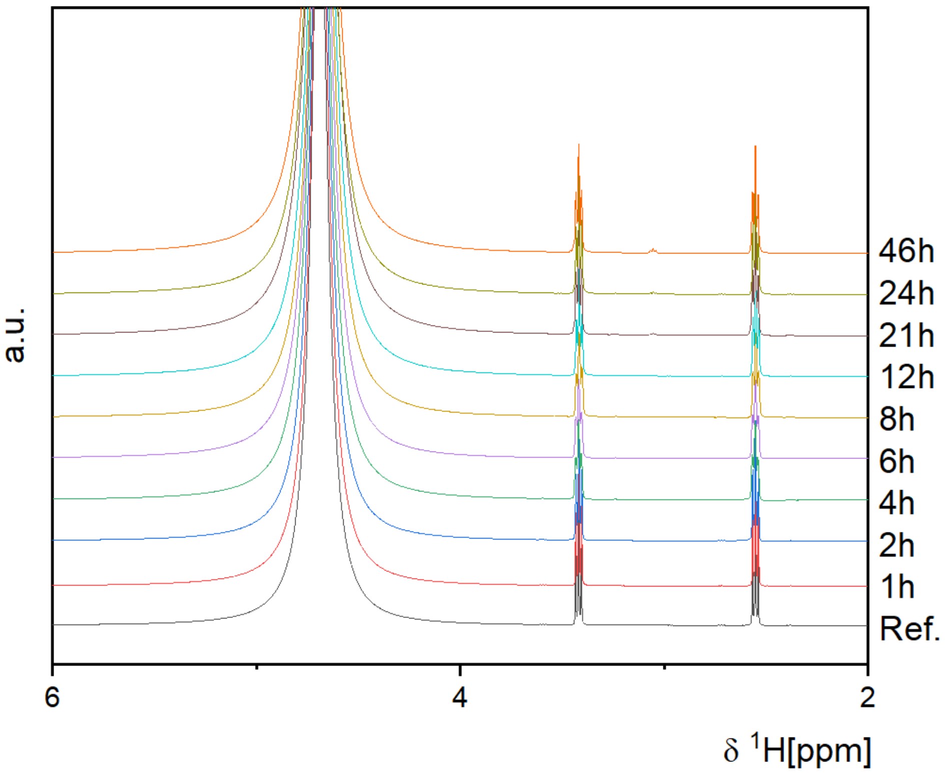

Figure S10. ^{1}H NMR spectra of experiment with 5% v/v ethanolamine in H_2O/D_2O with a ratio of ethanolamine:urea 1:6 at 50 °C.

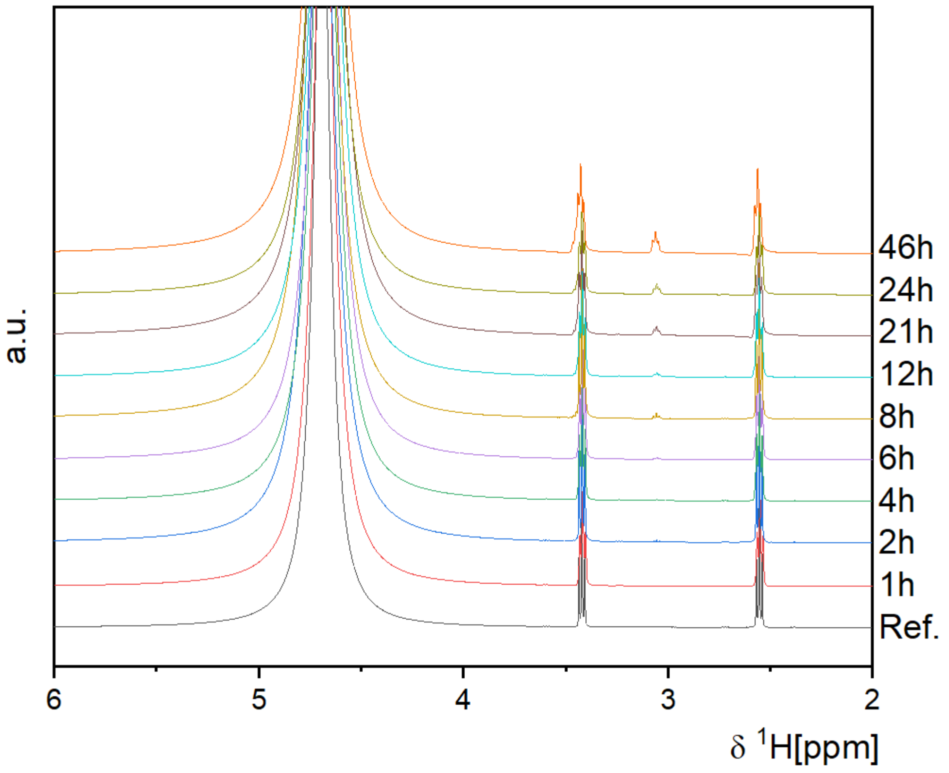

Figure S11. ^{1}H NMR spectra of experiment with 5% v/v ethanolamine in H_2O/D_2O with a ratio of ethanolamine:urea 1:6 at 60 °C.

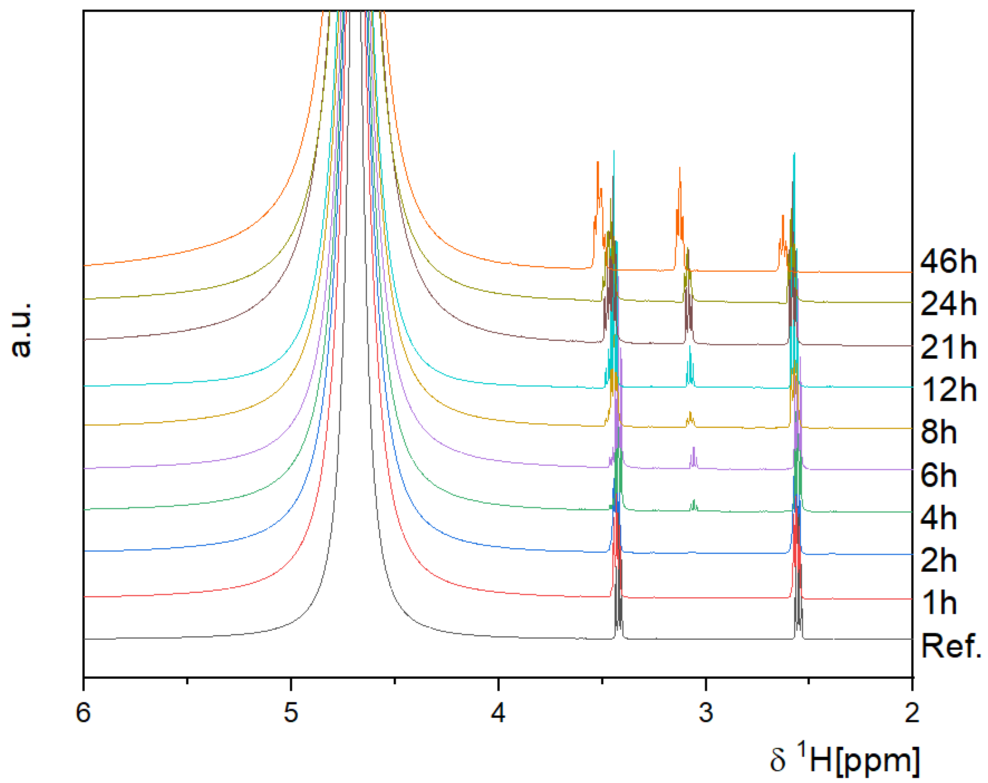

Figure S12. ^{1}H NMR spectra of experiment with 5% v/v ethanolamine in H_2O/D_2O with a ratio of ethanolamine:urea 1:6 at 70 °C.

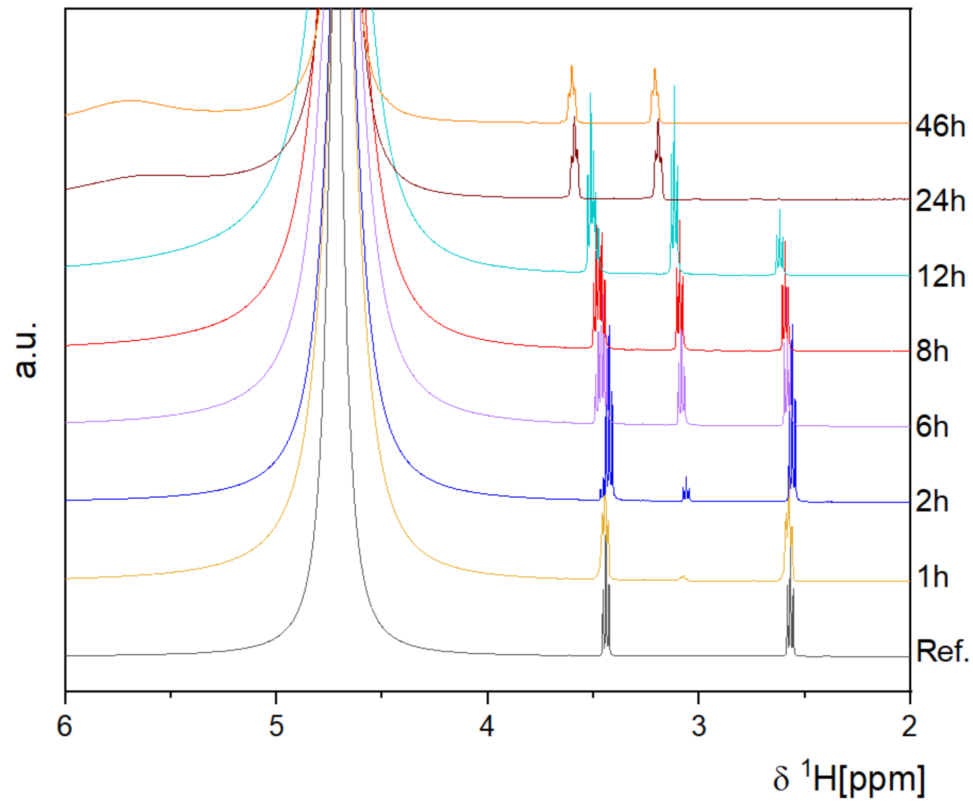

Figure S13. ^{1}H NMR spectra of experiment with 5% v/v ethanolamine in H_2O/D_2O with a ratio of ethanolamine:urea 1:6 at 80 °C.

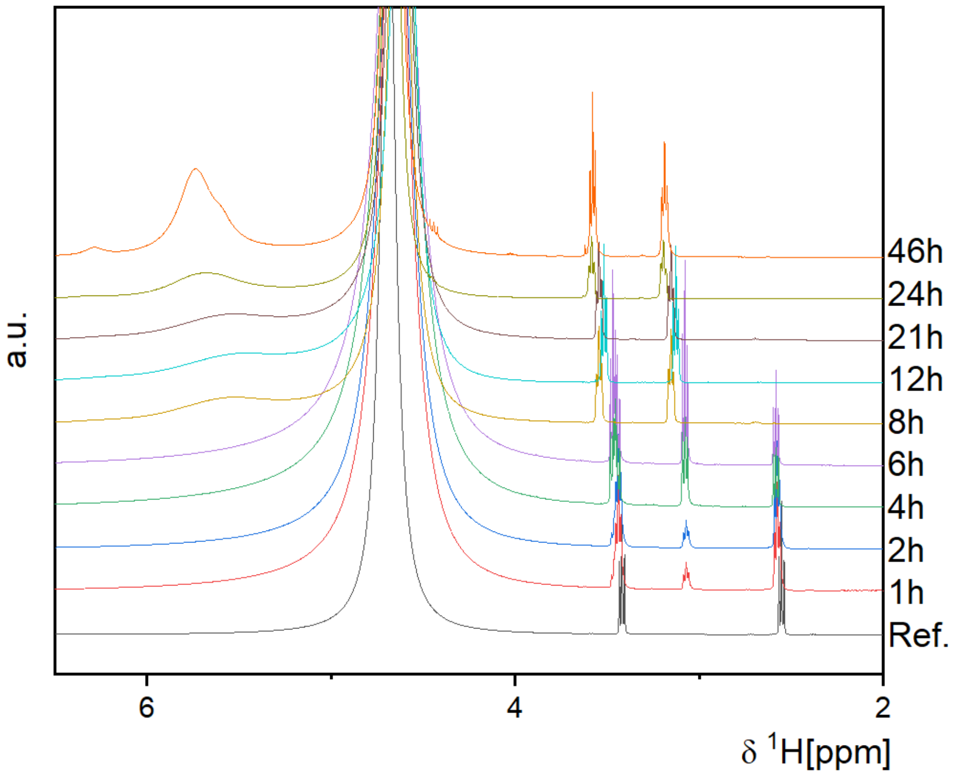

Figure S14. ^{1}H NMR spectra of experiment with 5% v/v ethanolamine in H_2O/D_2O with a ratio of ethanolamine:urea 1:6 at 90 °C.

Section S8. Effect of the solvent on ethanolamine:urea reaction

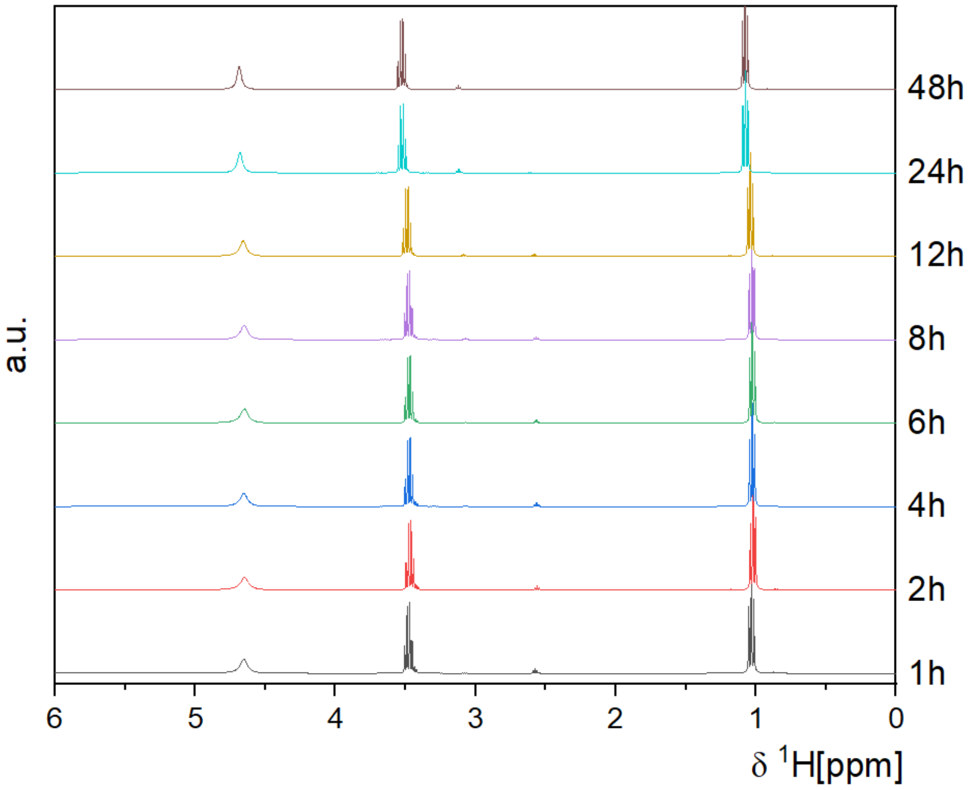

Figure S15. [1]*H NMR spectra of experiment with 5% v/v ethanolamine in 100% EtOH / 0%* H_2O *and a 6-fold urea excess (ethanolamine:urea = 1:6) at 80 °C.*

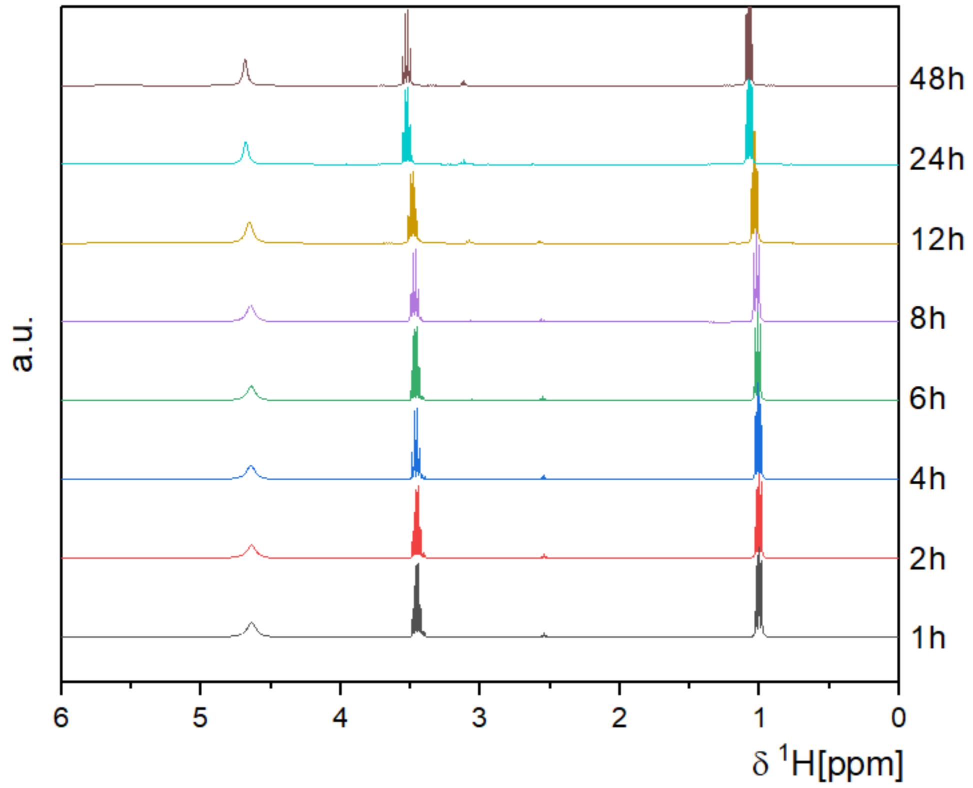

Figure S16. ¹H NMR spectra of experiment with 5% v/v ethanolamine in 98% EtOH / 2% H_2O and a 6-fold urea excess (ethanolamine:urea = 1:6) at 80 °C.

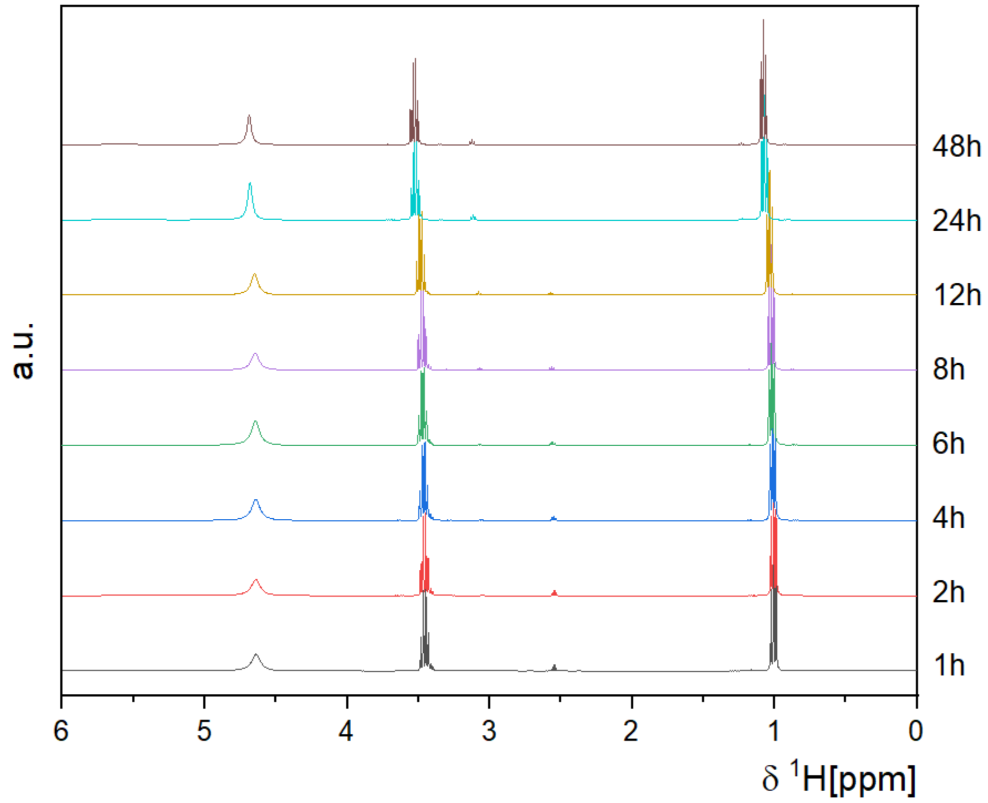

Figure S17. ¹H NMR spectra of experiment with 5% v/v ethanolamine in 95% EtOH / 5% H_2O and a 6-fold urea excess (ethanolamine:urea = 1:6) at 80 °C.

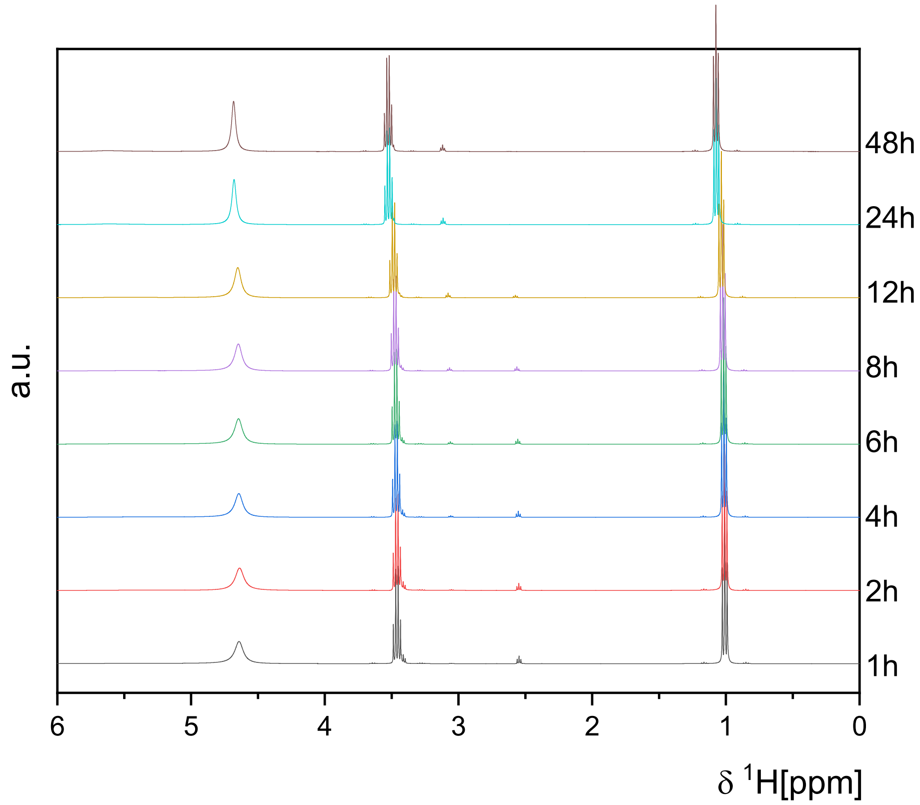

Figure S18. 1H NMR spectra of experiment with 5% v/v ethanolamine in 90% EtOH / 10% H_2O and a 6-fold urea excess (ethanolamine:urea = 1:6) at 80 °C.

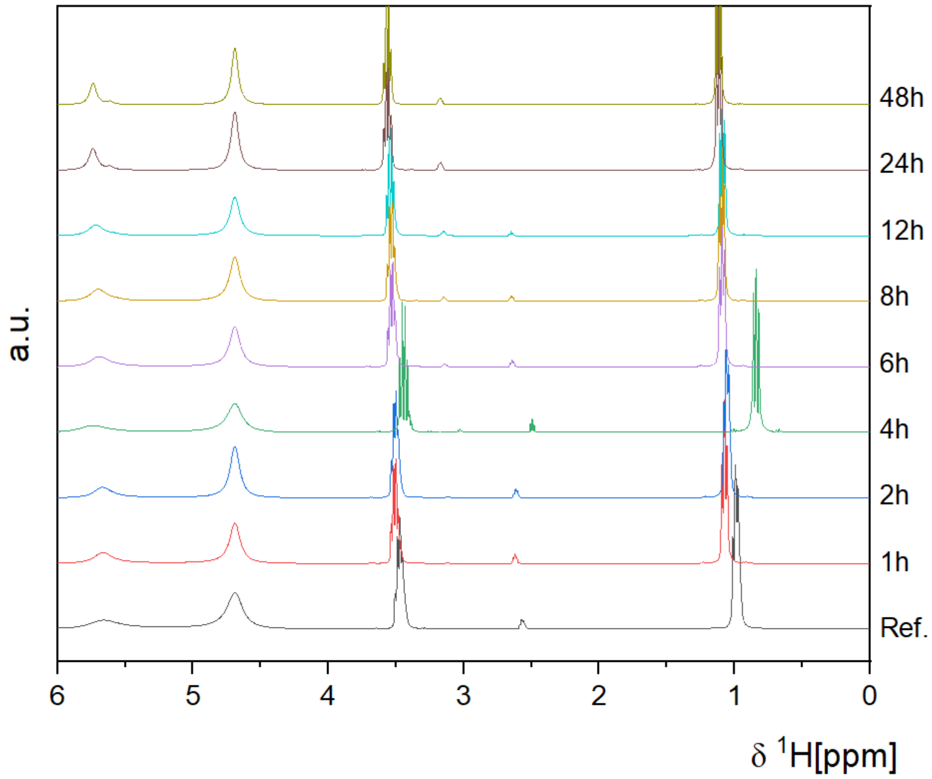

Figure S19. 1H NMR spectra of experiment with 5% v/v ethanolamine in 75% EtOH / 25% H_2O and a 6-fold urea excess (ethanolamine:urea = 1:6) at 80 °C.

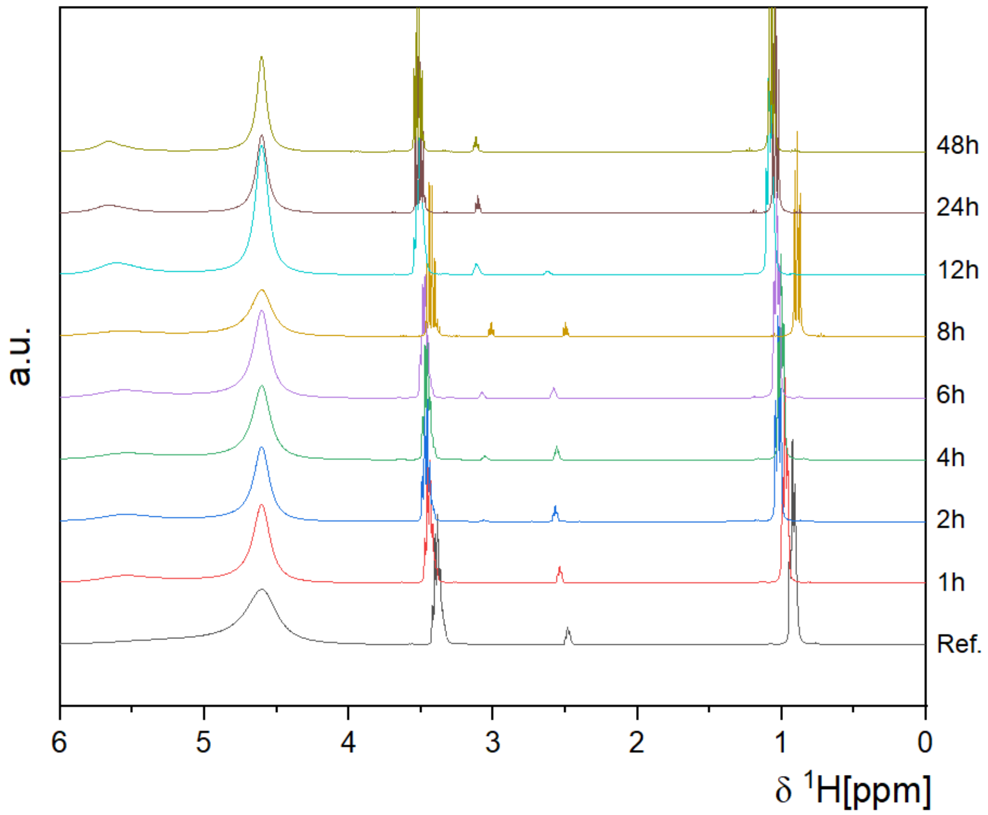

Figure S20. ¹H NMR spectra of experiment with 5% v/v ethanolamine in 50% EtOH / 50% H_2O and a 6-fold urea excess (ethanolamine:urea = 1:6) at 80 °C.

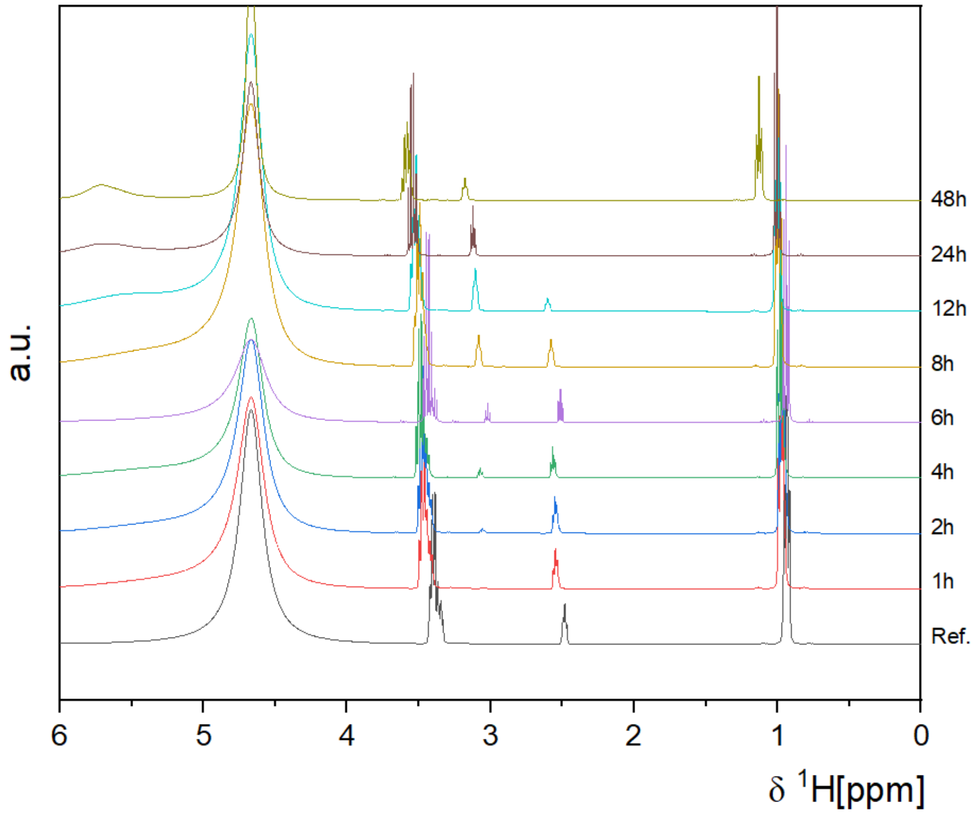

Figure S21. ¹H NMR spectra of experiment with 5% v/v ethanolamine in 25% EtOH / 75% H_2O and a 6-fold urea excess (ethanolamine:urea = 1:6) at 80 °C.

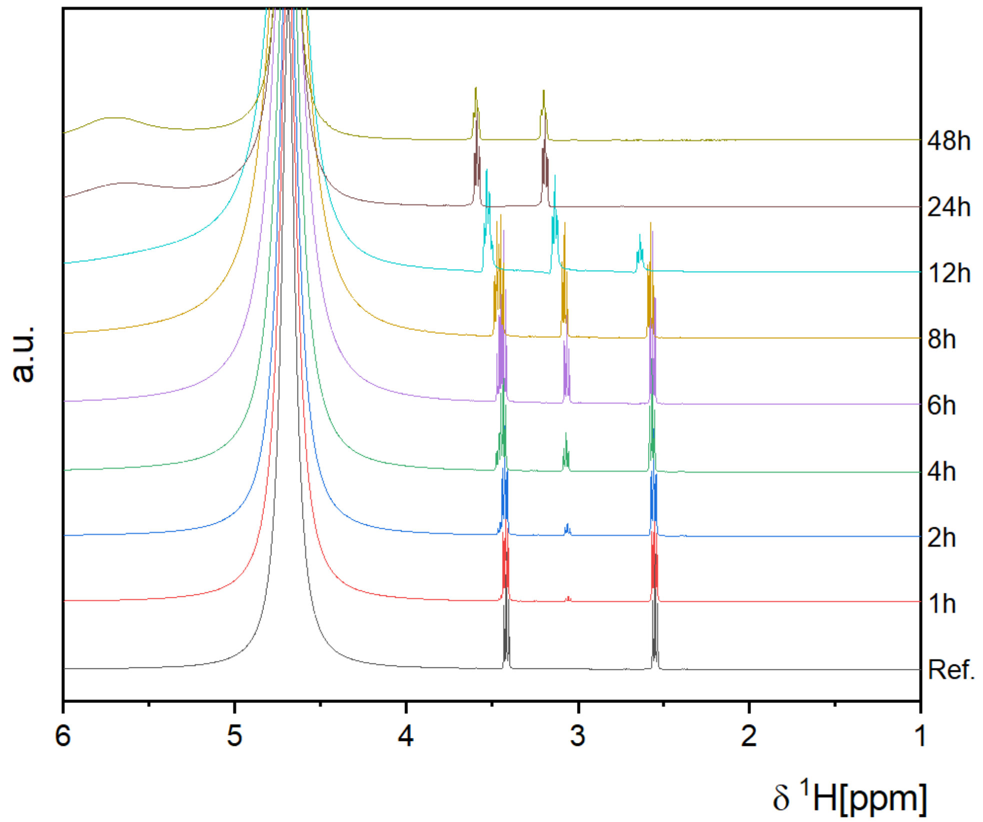

Figure S22. ^{1}H NMR spectra of experiment with 5% v/v ethanolamine in 0% EtOH/ 100% H_2O and a 6-fold urea excess (ethanolamine:urea = 1:6) at 80 °C.

Section S9. Kinetic model

The kinetic model is based on the following mechanism, discussed in the introduction of the manuscript:

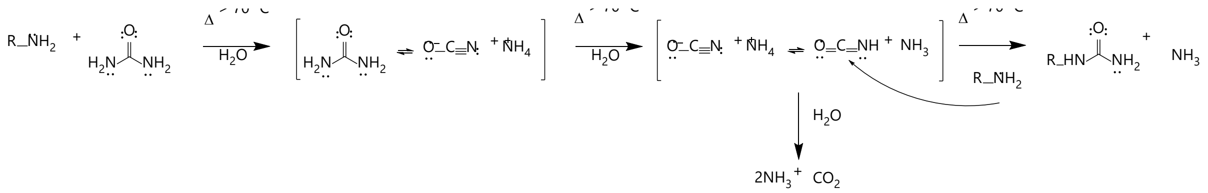

It is assumed that each reaction in the model is an elementary reaction. In the case of a primary amine, the equations are the following:

$$
\begin{aligned}
\frac{d\left[OC(NH_2)_2\right]}{dt} &= -k_1\left[OC(NH_2)_2\right]+k_{-1}[OCNH][NH_3] \\
\frac{d[OCNH]}{dt} &= k_1\left[OC(NH_2)_2\right]-k_{-1}[OCNH][NH_3]-k_2[OCNH][RNH_2]-k_4\left[OCN^-\right] \\
\frac{d[RNH_2]}{dt} &= -k_2[OCNH][RNH_2] \\
\frac{d[NH_3]}{dt} &= k_1\left[OC(NH_2)_2\right]-k_{-1}[OCNH][NH_3]+k_2[OCNH][RNH_2]+k_4\left[OCN^-\right] \\
\frac{d[RNHCONH_2]}{dt} &= k_2[OCNH][RNH_2]-k_3[RNHCONH_2] \\
\frac{d\left[HCO_3^-\right]}{dt} &= +k_4\left[OCN^-\right]
\end{aligned}
\tag{0.1}
$$

In addition to the kinetic equations, all acid-base equilibria must be used to relate the concentration of ions to those of undissociated species, in addition to the electroneutrality condition:

$$
\begin{aligned}
&K_a=\frac{\left[OCN^-\right]\left[H^+\right]}{[OCNH]};K_{b2}=\frac{\left[RNH_3^+\right]\left[OH^-\right]}{[RNH_2]};K_b=\frac{\left[NH_4^+\right]\left[OH^-\right]}{[NH_3]};K_w=\left[H^+\right]\left[OH^-\right] \\
&K_{ac2}=\frac{\left[CO_3^{2-}\right]\left[H^+\right]}{\left[HCO_3^-\right]};K_{ac1}=\frac{\left[HCO_3^-\right]\left[H^+\right]}{[H_2CO_3]};K_h=\frac{[H_2CO_3]}{[CO_2]_{aq}} \\
&\left[OCN^-\right]+\left[OH^-\right]+2\left[CO_3^{2-}\right]+\left[HCO_3^-\right]=\left[NH_4^+\right]+\left[RNH_3^+\right]+\left[H^+\right]
\end{aligned}
\tag{0.2}
$$

After some algebraic manipulations, the equilibrium relations can be combined to the kinetic equations as follows:

$$\begin{cases} \dfrac{d\left[OC(NH_2)_2\right]}{dt} = -k_1\left[OC(NH_2)_2\right] + k_{-1}\left[NH_3\right]_0\left(1-\dfrac{K_b}{K_b+\left[OH^-\right]}\right)\left[OCNH\right]_0\left(1-\dfrac{K_a\left[OH^-\right]}{K_w+K_a\left[OH^-\right]}\right) \\ \dfrac{d\left[OCNH\right]_0}{dt} = k_1\left[OC(NH_2)_2\right] - \left[OCNH\right]_0\left(1-\dfrac{K_a\left[OH^-\right]}{K_w+K_a\left[OH^-\right]}\right)\cdot \\ \left(k_{-1}\left[NH_3\right]_0\left(1-\dfrac{K_b}{K_b+\left[OH^-\right]}\right)+k_2\left[RNH_2\right]_0\left(1-\dfrac{K_{b2}}{\left[OH^-\right]+K_{b2}}\right)\right) - k_4\dfrac{K_a\left[OH^-\right]\left[OCNH\right]_0}{K_w+K_a\left[OH^-\right]} \\ \dfrac{d\left[RNH_2\right]_0}{dt} = -k_2\left[OCNH\right]_0\left(1-\dfrac{K_a\left[OH^-\right]}{K_w+K_a\left[OH^-\right]}\right)\left[RNH_2\right]_0\left(1-\dfrac{K_{b2}}{\left[OH^-\right]+K_{b2}}\right) \\ \dfrac{d\left[NH_3\right]}{dt} = k_1\left[OC(NH_2)_2\right] + \left[OCNH\right]_0\left(1-\dfrac{K_a\left[OH^-\right]}{K_w+K_a\left[OH^-\right]}\right)\cdot \\ \left(-k_{-1}\left[NH_3\right]_0\left(1-\dfrac{K_b}{K_b+\left[OH^-\right]}\right)+k_2\left[RNH_2\right]_0\left(1-\dfrac{K_{b2}}{\left[OH^-\right]+K_{b2}}\right)\right) + k_4\dfrac{K_a\left[OH^-\right]\left[OCNH\right]_0}{K_w+K_a\left[OH^-\right]} \\ \dfrac{d\left[RNHCONH_2\right]}{dt} = k_2\left[OCNH\right]_0\left(1-\dfrac{K_a\left[OH^-\right]}{K_w+K_a\left[OH^-\right]}\right)\left[RNH_2\right]_0\left(1-\dfrac{K_{b2}}{\left[OH^-\right]+K_{b2}}\right) - k_3\left[RNHCONH_2\right] \\ \dfrac{d\left[HCO_3^-\right]_0}{dt} = +k_4\dfrac{K_a\left[OH^-\right]\left[OCNH\right]_0}{K_w+K_a\left[OH^-\right]} \end{cases} \quad (0.3)$$

Where the concentration of hydroxyl ions can be found from the electroneutrality equation, which is written as follows:

$$\frac{K_a\left[OH^-\right]\left[OCNH\right]_0}{K_w+K_a\left[OH^-\right]} + \left[OH^-\right] + \left[HCO_3^-\right]_0\left(1-\frac{K_w+\left[OH^-\right]^2\dfrac{K_{ac2}K_{ac1}K_h}{(1+K_h)K_w}}{\left(K_{ac1}-\dfrac{K_{ac1}}{1+K_h}\right)\left[OH^-\right]+K_w+\left[OH^-\right]^2\dfrac{K_{ac2}K_{ac1}K_h}{(1+K_h)K_w}}\right) \quad (0.4)$$

$$+2\frac{\left[HCO_3^-\right]_0\left[OH^-\right]^2\dfrac{K_{ac2}K_{ac1}K_h}{(1+K_h)K_w}}{\left(K_{ac1}-\dfrac{K_{ac1}}{1+K_h}\right)\left[OH^-\right]+K_w+\left[OH^-\right]^2\dfrac{K_{ac2}K_{ac1}K_h}{(1+K_h)K_w}} = \frac{\left[NH_3\right]_0K_b}{K_b+\left[OH^-\right]} + \frac{\left[RNH_2\right]_0K_{b2}}{\left[OH^-\right]+K_{b2}} + \frac{K_w}{\left[OH^-\right]}$$

The above algebraic equation must be numerically solved at every integration step. In the manner, the pH of the solution can be also calculated immediately as a function of time. The reaction of consumption of ureido has been introduced in order to explain the disappearance of ureido in the very long-term kinetic experiment of consumption of ethanolamine.

In the case of a glycine, which is an amino acid, the ionic equilibria are more complex, because of the additional dissociation reactions:

$$K_{am1} = \frac{\left[CO_2^-CH_2NH_3^+\right]K_w}{\left[CO_2HCH_2NH_3^+\right]\left[OH^-\right]};K_{am2} = \frac{\left[CO_2^-CH_2NH_2\right]K_w}{\left[CO_2^-CH_2NH_3^+\right]\left[OH^-\right]};K_{am3} = \frac{\left[CO_2^-CH_2NH_2\right]K_w}{\left[CO_2HCH_2NH_2\right]\left[OH^-\right]} \quad (0.5)$$

Even the corresponding ureido, which is hydantoic acid, has a dissociation equilibrium:

$$K_{a3} = \frac{\left[CO_2^- CH_2NHCONH_2\right] K_w}{\left[CO_2HCH_2NHCONH_2\right]\left[OH^-\right]} \tag{0.6}$$

Therefore, the electroneutrality condition is still In the case of a diamine, there are two dissociation equilibria:

$$K_{b2} = \frac{\left[NH_2RNH_3^+\right]\left[OH^-\right]}{\left[NH_2RNH_2\right]}; K_{b3} = \frac{\left[NH_3RNH_3^{2+}\right]\left[OH^-\right]}{\left[NH_2RNH_3^+\right]} \tag{0.7}$$

The kinetic equations become:

$$\begin{cases}
\frac{d\left[OC(NH_2)_2\right]}{dt} = -k_1\left[OC(NH_2)_2\right] + k_{-1}\left[NH_3\right]_0\left(1-\frac{K_b}{K_b+\left[OH^-\right]}\right)\left[OCNH\right]_0\left(1-\frac{K_a\left[OH^-\right]}{K_w+K_a\left[OH^-\right]}\right) \\
\frac{d\left[OCNH\right]_0}{dt} = k_1\left[OC(NH_2)_2\right] - k_{-1}\left[OCNH\right]_0\left(1-\frac{K_a\left[OH^-\right]}{K_w+K_a\left[OH^-\right]}\right)\cdot \\
\left(k_{-1}\left[NH_3\right]_0\left(1-\frac{K_b}{K_b+\left[OH^-\right]}\right) + k_2\left[RNH_2\right]_0\left(1-\frac{K_{b2}}{\left[OH^-\right]+K_{b2}}\right) - \alpha k_2\left[NH_2RNHCONH_2\right]_0\left(1-\frac{K_{b2}}{\left[OH^-\right]+K_{b2}}\right)\right) - k_4\frac{K_a\left[OH^-\right]\left[OCNH\right]_0}{K_w+K_a\left[OH^-\right]} \\
\frac{d\left[NH_2RNH_2\right]_0}{dt} = -k_2\left[OCNH\right]_0\left(1-\frac{K_a\left[OH^-\right]}{K_w+K_a\left[OH^-\right]}\right)\left[NH_2RNH_2\right]_0\left(1-\frac{\left(\left[OH^-\right]K_{b2}+K_{b3}K_{b2}\right)}{\left[OH^-\right]^2+\left[OH^-\right]K_{b2}+K_{b3}K_{b2}}\right) \\
\frac{d\left[NH_3\right]}{dt} = k_1\left[OC(NH_2)_2\right] + \left[OCNH\right]_0\left(1-\frac{K_a\left[OH^-\right]}{K_w+K_a\left[OH^-\right]}\right)\cdot \\
\left(\begin{array}{l} -k_{-1}\left[NH_3\right]_0\left(1-\frac{K_b}{K_b+\left[OH^-\right]}\right) + k_2\left[NH_2RNH_2\right]_0\left(1-\frac{\left(\left[OH^-\right]K_{b2}+K_{b3}K_{b2}\right)}{\left[OH^-\right]^2+\left[OH^-\right]K_{b2}-K_{b3}K_{b2}}\right) \\ +\alpha k_2\left[NH_2RNHCONH_2\right]_0\left(1-\frac{K_{b2}}{\left[OH^-\right]+K_{b2}}\right) \end{array}\right) + k_4\frac{K_a\left[OH^-\right]\left[OCNH\right]_0}{K_w+K_a\left[OH^-\right]} \\
\frac{d\left[NH_2RNHCONH_2\right]_0}{dt} = k_2\left[OCNH\right]_0\left(1-\frac{K_a\left[OH^-\right]}{K_w+K_a\left[OH^-\right]}\right)\left[NH_2RNH_2\right]_0\left(1-\frac{\left(\left[OH^-\right]K_{b2}+K_{b3}K_{b2}\right)}{\left[OH^-\right]^2+\left[OH^-\right]K_{b2}+K_{b3}K_{b2}}\right) - \\
\alpha k_2\left[OCNH\right]_0\left(1-\frac{K_a\left[OH^-\right]}{K_w+K_a\left[OH^-\right]}\right)\left[NH_2RNHCONH_2\right]_0\left(1-\frac{K_{b2}}{\left[OH^-\right]+K_{b2}}\right) \\
\frac{d\left[NH_2CONHRNHCONH_2\right]}{dt} = \alpha k_2\left[OCNH\right]_0\left(1-\frac{K_a\left[OH^-\right]}{K_w+K_a\left[OH^-\right]}\right)\left[NH_2RNHCONH_2\right]_0\left(1-\frac{K_{b2}}{\left[OH^-\right]+K_{b2}}\right) \\
\frac{d\left[HCO_3^-\right]_0}{dt} = +k_4\frac{K_a\left[OH^-\right]\left[OCNH\right]_0}{K_w+K_a\left[OH^-\right]}
\end{cases} \tag{0.8}$$

The factor α was introduced to account for the different reactivity of the second amidation reaction. It was found by fitting the experimental data, that α is equal to about 0.5, in fairly close agreement with the simple Monte-Carlo calculation reported in the paper.

The last case is the triamine. The complexity in this case is related to the many intermediate species that can be formed. The triamine has three dissociation equilibria:

$$K_{b2} = \frac{\left[H_2NRNHRNH_3^+\right]\left[OH^-\right]}{\left[H_2NRNHRNH_2\right]}; K_{b3} = \frac{\left[H_3NRNHRNH_3^{2+}\right]\left[OH^-\right]}{\left[H_2NRNHRNH_3^+\right]}; K_{b4} = \frac{\left[H_3NRNH_2RNH_3^{3+}\right]\left[OH^-\right]}{\left[H_3NRNHRNH_3^{2+}\right]}$$

Accounting for all of them, we have the following:

$$
\begin{cases}
\frac{d\left[OC\left(NH_2\right)_2\right]}{dt} = -k_1\left[OC\left(NH_2\right)_2\right] + k_{-1}\left[NH_3\right]\left[OCNH\right] \\
\frac{d\left[OCNH\right]_0}{dt} = k_1\left[OC\left(NH_2\right)_2\right] - \left[OCNH\right]\begin{pmatrix} k_{-1}\left[NH_3\right] + 3k_2\left[H_2NRNHRNH_2\right] + 2k_2\left[OCNH_2RNHRNH_2\right] \\ +2k_2\left[H_2NRCONH_2RNH_2\right] + k_2\left[CONH_2RCONH_2RNH_2\right] + \\ k_2\left[OCNH_2RNHROCNH_2\right] \end{pmatrix} \\
-k_4\frac{K_a\left[OH^-\right]\left[OCNH\right]_0}{K_w + K_a\left[OH^-\right]} \\
\frac{d\left[H_2NRNHRNH_2\right]_0}{dt} = -3k_2\left[OCNH\right]\left[H_2NRNHRNH_2\right] \\
\frac{d\left[OCNH_2RNHRNH_2\right]}{dt} = 2k_2\left[OCNH\right]\left[H_2NRNHRNH_2\right] - 2k_2\left[OCNH\right]\left[OCNH_2RNHRNH_2\right] \\
\frac{d\left[H_2NRCONH_2RNH_2\right]}{dt} = k_2\left[OCNH\right]\left[H_2NRNHRNH_2\right] - 2k_2\left[OCNH\right]\left[H_2NRCONH_2RNH_2\right] \\
\frac{d\left[CONH_2RCONH_2RNH_2\right]}{dt} = 2k_2\left[OCNH\right]\left[H_2NRCONH_2RNH_2\right] + k_2\left[OCNH\right]\left[OCNH_2RNHRNH_2\right] \\
-k_2\left[OCNH\right]\left[CONH_2RCONH_2RNH_2\right] \\
\frac{d\left[OCNH_2RNHROCNH_2\right]}{dt} = k_2\left[OCNH\right]\left[OCNH_2RNHRNH_2\right] - k_2\left[OCNH\right]\left[OCNH_2RNHROCNH_2\right] \\
\frac{d\left[OCNH_2RCONH_2ROCNH_2\right]}{dt} = k_2\left[OCNH\right]\left[OCNH_2RNHROCNH_2\right] + k_2\left[OCNH\right]\left[CONH_2RCONH_2RNH_2\right] \\
\frac{d\left[NH_3\right]}{dt} = k_1\left[OC\left(NH_2\right)_2\right] + \left[OCNH\right]\begin{pmatrix} -k_{-1}\left[NH_3\right] + 3k_2\left[H_2NRNHRNH_2\right] + 2k_2\left[H_2NRCONH_2RNH_2\right] \\ +2k_2\left[OCNH\right]\left[OCNH_2RNHRNH_2\right] + k_2\left[OCNH\right]\left[CONH_2RCONH_2RNH_2\right] \\ +k_2\left[OCNH\right]\left[OCNH_2RNHROCNH_2\right] \end{pmatrix} \\
+k_4\frac{K_a\left[OH^-\right]\left[OCNH\right]_0}{K_w + K_a\left[OH^-\right]} \\
\frac{d\left[HCO_3^-\right]_0}{dt} = +k_4\frac{K_a\left[OH^-\right]\left[OCNH\right]_0}{K_w + K_a\left[OH^-\right]}
\end{cases}
\tag{0.9}
$$

The assumption made in Table S3 contains the values of the various kinetic and equilibrium constants used in the simulations, with the corresponding references where they were found. In some cases, instead of values, an expression providing a function of the temperature has been reported. Table S4 instead shows the values of the kinetic constant k_2 obtained from the various amines, and in the case of ethanolamine at different temperatures.

Table S3. Equilibrium constants and kinetic constants used in the simulations

Constant	Expression or value	Reference
K_a	$2.1\cdot10^{-4}\cdot exp(5400/R\cdot(1/T-1/298.15))$ [Mol/L]	1
K_w	$exp(148.9802 - 13847.26/T - 23.6521\cdot log(T))$ [Mol²/L²]	2
K_{ac1}	$10^{-(3404.71/T + 0.032786- 14.8435)}$ [Mol/L]	2
K_{ac2}	$10^{-(2902.39/T + 0.02379- 6.4980)}$ [Mol/L]	2
K_h	$10^{(2622.38/T+0.0178471T -15.5873)}$	2
K_{b2} *(ethanolamine)*	$K_W\cdot exp(53900/R/T)$ [Mol/L]	3
K_b *(ammonia)*	$K_w\cdot10^{10.0423-(0.0315536*(T-273.15))}$ [Mol/L]	4
K_{b2} *(5-amino 1-pentanol)*	$3.625\cdot10^{-5}$ [Mol/L]	5
K_{am1}	0.0049 [Mol/L]	6
K_{am2}	$2.3988\cdot10^{-10}$ [Mol/L]	6
K_{am3}	$4.8978\cdot10^{-5}$ [Mol/L]	6
K_a *(hydantoic acid)*	$1.3305\cdot10^{-4}$ [Mol/L]	7
K_{b2} *(N,N´-dimethyl-1,3-propanediamine)*	$4.1687\cdot10^{-4}$ [Mol/L]	7
K_{b3} *(N,N´-dimethyl-1,3-propanediamine)*	$1.9953\cdot10^{-11}$ [Mol/L]	7
K_{b2} *(hexamethylendiamine)*	$5.0119\cdot10^{-4}$ [Mol/L]	8
K_{b3} *(hexamethylendiamine)*	$1.9953\cdot10^{-11}$ [Mol/L]	8
K_{b2} *(bis-(Hexamethylene)triamine)*	$5.0119\cdot10^{-4}$ [Mol/L]	7
K_{b3} *(bis-(Hexamethylene)triamine)*	$1.9953\cdot10^{-11}$ [Mol/L]	7
K_{b4} *(bis-(Hexamethylene)triamine)*	10^{-13} [Mol/L]	7
k_1	$4\cdot10^{14}\cdot exp(-135560/8.314/T)$ [s^{-1}]	9
k_3	$1.017\cdot10^{-7}$ [s^{-1}]	Fitted
k_4	$7.2\cdot10^{8}\cdot exp(-10900/T)$ [L/Mol·s]	1
k_{-1}	$10^{11}\cdot exp(-95718/R/T)\cdot K_aK_b/K_w$ [L/Mol·s]	10

Table S4. Fitted values of k_2 rate constants for the reaction of isocyanic acid with various amines

k_2 *ethanolamine, 90°C*	278 [L/Mol/s]
k_2 *ethanolamine, 80°C*	278 [L/Mol/s]
k_2 *ethanolamine, 70°C*	278 [L/Mol/s]
k_2 *ethanolamine, 60°C*	278 [L/Mol/s]
k_2 *ethanolamine, 50°C*	278 [L/Mol/s]
k_2 *5-amino 1-pentanol, 80°C*	2566 [L/Mol/s]
k_2 *glycine, 80°C*	2762710 [L/Mol/s]
k_2 *N,N´-dimethyl-1,3-propanediamine, 80°C*	2279 [L/Mol/s]
k_2 *hexamethylenediamine, 80°C*	5093 [L/Mol/s]
k_2 *bis-(hexamethylene)triamine, 80°C*	1216 [L/Mol/s]

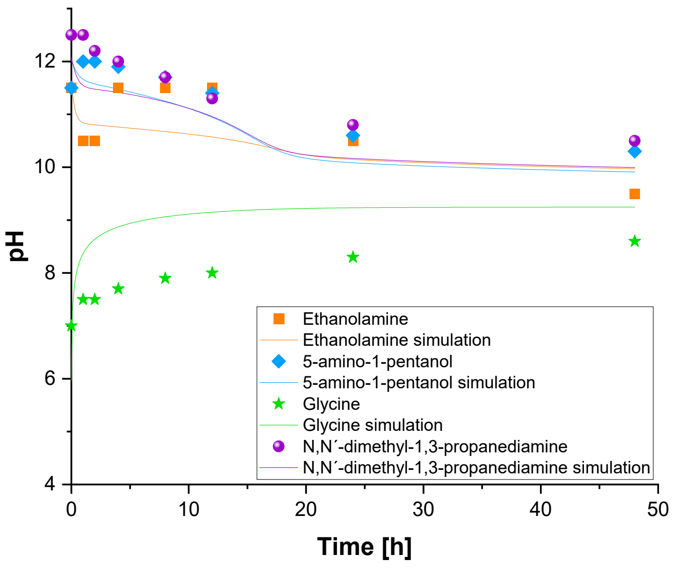

Figure S23. pH variation as a function of reaction time for different substrates. The fitted lines correspond to the results of the kinetic modelling (See section 3.5). The reactions were conducted at 80 °C with 5% v/v of amine molecule in H_2O/D_2O and with a 6-fold urea excess: ethanolamine : urea = 1:6, 5-amino-1-pentanol : urea = 1:6, glycine : urea = 1:6, N,N'-dimethyl-1,3-propanediamine:urea = 1:12.

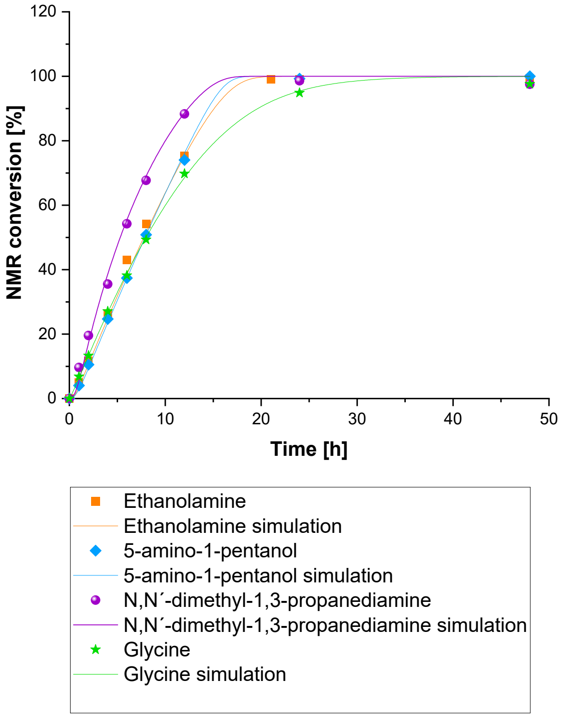

Figure S24. Ureido NMR conversion as a function of reaction time for different substrates. The reactions were conducted at 80 °C with 5% v/v of amine molecule in H_2O/D_2O and with a 6-fold urea excess: ethanolamine : urea = 1:6, 5-amino-1-pentanol : urea = 1:6, N,N'-dimethyl-1,3-propanediamine:urea = 1:12, glycine : urea = 1:6. The fitted lines correspond to the results of the kinetic modelling (See section 3.5).

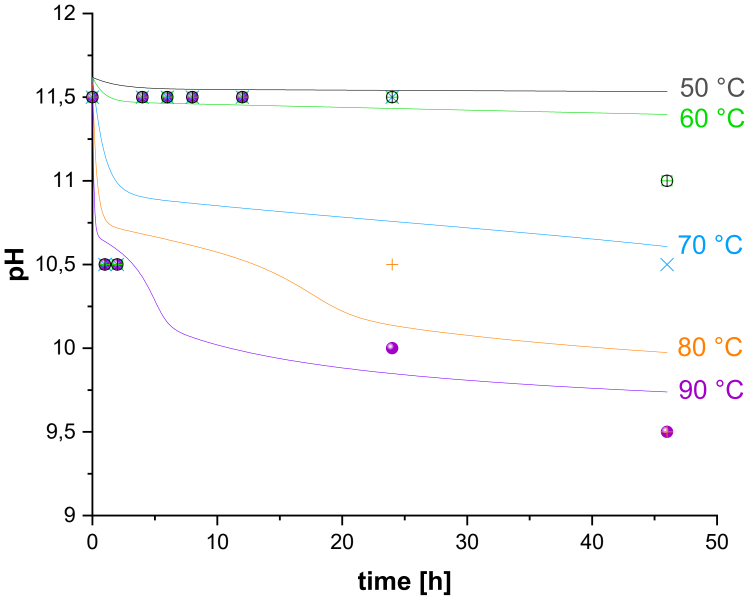

Figure S25. Time dependent pH variation as a function of reaction temperature. The reactions were conducted with 5%v/v of ethanolamine in H_2O/D_2O with a 6-fold excess of urea (ethanolamine : urea = 1:6). The fitted lines correspond to the results of the kinetic modelling (See section 3.5).

Section S10. ^{13}C NMR spectra

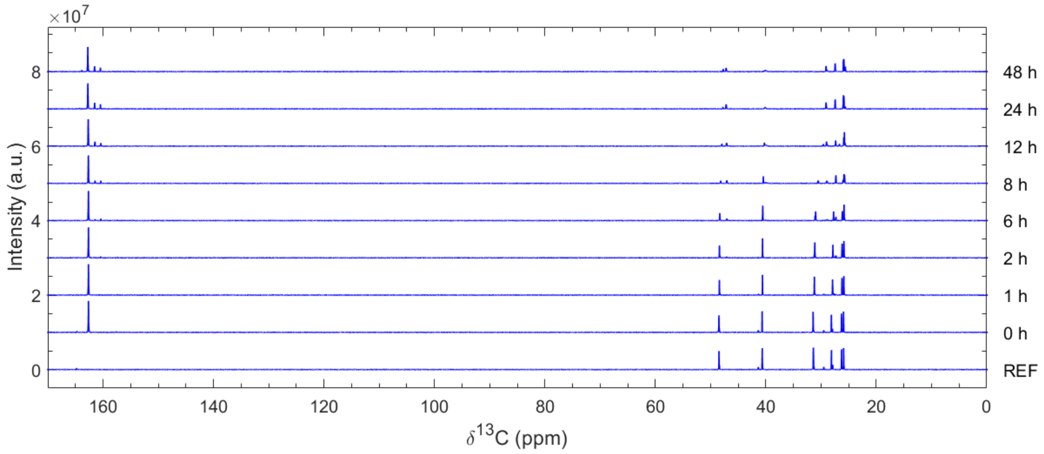

Figure S26. ^{13}C NMR spectra of the possible reaction products of bis-(hexamethylene)triamine with urea. The reaction was conducted at 80 °C with 5%v/v of bis-(hexamethylene)triamine in H_2O/D_2O and with a 18-fold urea excess with respect to the urea:amine ratio, corresponding to (bis-(hexamethylene)triamine:urea 1:18).

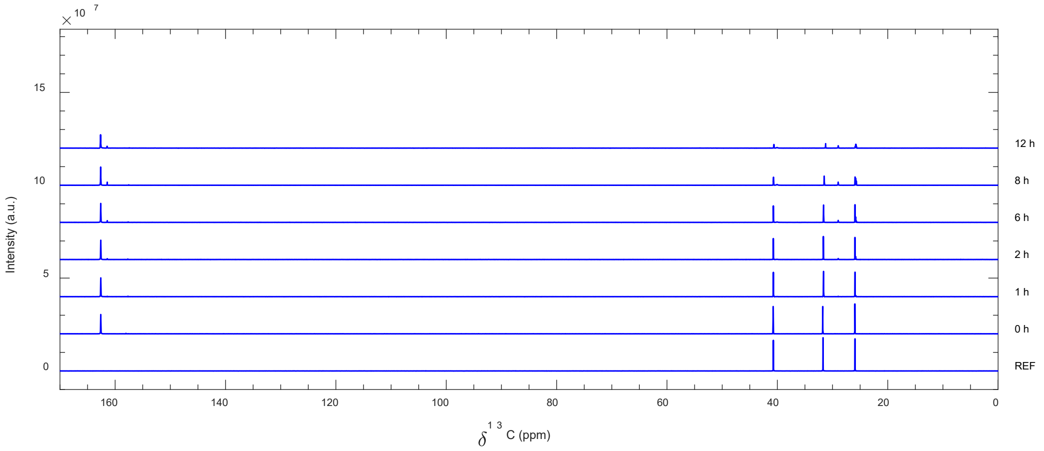

Figure S27. ^{13}C NMR spectra of the products of hexamethylenediamine after reaction with urea. The reaction was conducted at 80 °C with 5%v/v of hexamethylenediamine in H_2O/D_2O and with a 12-fold urea excess (hexamethylene-diamine:urea 1:12).

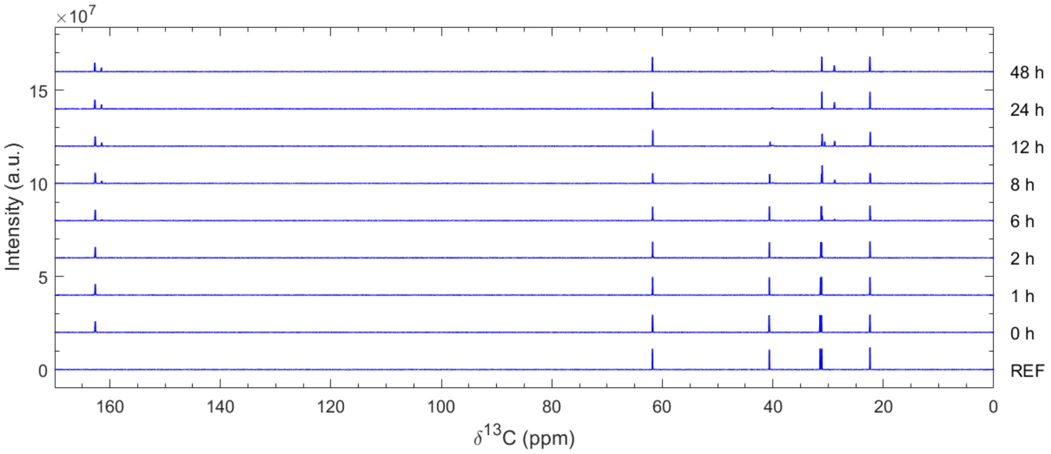

Figure S 28. ^{13}C *NMR spectra and numbering of positions of the possible reaction products of 5-amino-1-pentanol after reaction with urea. The reaction was conducted at 80 °C with 5%v/v of 5-amino-1-pentanol in* H_2O/D_2O *and with a 6-fold urea excess (5-amino-1-pentanol:urea 1:6).*

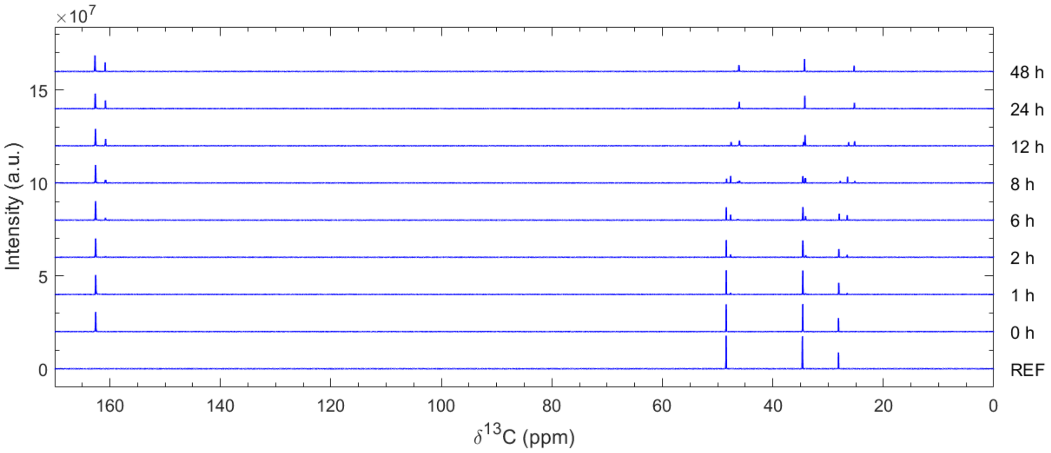

Figure S29. . ^{13}C *NMR spectra of the possible reaction products of N,N'-dimethyl-1,3-propanediamine after reaction with urea. The reaction was conducted at 80 °C with 5%v/v of N,N'-dimethyl-1,3-propanediamine in* H_2O/D_2O *and with a 12-fold urea excess (N,N'-dimethyl-1,3-propanediamine:urea 1:12).*

References

(1) Borduas, N.; Place, B.; Wentworth, G. R.; Abbatt, J. P. D.; Murphy, J. G. Solubility and Reactivity of HNCO in Water: Insights into HNCO's Fate in the Atmosphere. *Atmos. Chem. Phys.* **2016**, *16* (2), 703–714. https://doi.org/10.5194/acp-16-703-2016.

(2) Mook, W. G. *Environmental Isotopes in the Hydrological Cycle: Principles and Applications, Volume I: Introduction: Theory, Methods, Review*; 2001; Vol. 1.

(3) Hamborg, E. S.; Versteeg, G. F. Dissociation Constants and Thermodynamic Properties of Amines and Alkanolamines from (293 to 353) K. *J. Chem. Eng. Data* **2009**, *54* (4), 1318–1328. https://doi.org/10.1021/je800897v.

(4) Bell, T. G.; Johnson, M. T.; Jickells, T. D.; Liss, P. S. Ammonia/Ammonium Dissociation Coefficient in Seawater: A Significant Numerical Correction. *Environ. Chem.* **2007**, *4* (3), 183. https://doi.org/10.1071/EN07032.

(5) Hesse, M. Indolalkaloide in Tabellen. *J. Am. Chem. Soc.* **1964**, *86* (24), 5706–5707. https://doi.org/10.1021/ja01078a084.

(6) Scholz, F.; Kahlert, H. Acid–Base Equilibria of Amino Acids: Microscopic and Macroscopic Acidity Constants. *ChemTexts* **2018**, *4* (2), 6. https://doi.org/10.1007/s40828-018-0060-5.

(7) Inc., C. Chemical Book https://www.chemicalbook.com/ (accessed Dec 7, 2020).

(8) Perrin, D. D.; Dempsey, B. Buffers for PH and Metal Ion Control. *J. AOAC Int.* **1975**, *58* (4), 864–864. https://doi.org/10.1093/jaoac/58.4.864.

(9) Shaw, W. H. R.; Bordeaux, J. J. The Decomposition of Urea in Aqueous Media. *J. Am. Chem. Soc.* **1955**, *77* (18), 4729–4733. https://doi.org/10.1021/ja01623a011.

(10) Welles, H. L.; Giaquinto, A. R.; Lindstrom, R. E. Degradation of Urea in Concentrated Aqueous Solution. *J. Pharm. Sci.* **1971**, *60* (8), 1212–1216. https://doi.org/10.1002/jps.2600600820.
